# Alzheimer's disease brain endothelial-like cells reveal differential drug transporter expression and modulation by potentially therapeutic focused ultrasound

**DOI:** 10.1016/j.neurot.2023.10.009

**Published:** 2023-12-19

**Authors:** Juliana C.S. Chaves, Joanna M. Wasielewska, Carla Cuní-López, Laura M. Rantanen, Serine Lee, Jari Koistinaho, Anthony R. White, Lotta E. Oikari

**Affiliations:** a Mental Health and Neuroscience, QIMR Berghofer Medical Research Institute, Brisbane, QLD, Australia; b School of Biomedical Sciences, Faculty of Health, Queensland University of Technology, QUT, Brisbane, QLD, Australia; c Faculty of Medicine, The University of Queensland, Brisbane, QLD, Australia; d A.I. Virtanen Institute for Molecular Sciences, University of Eastern Finland, Neuroscience Center, Kuopio, Finland; e Helsinki Institute of Life Science, University of Helsinki, Helsinki, Finland

**Keywords:** Alzheimer's disease, Blood-brain barrier, Induced pluripotent stem cells, Transporters, Ultrasound therapy, Drug transport

## Abstract

The blood-brain barrier (BBB) has a key function in maintaining homeostasis in the brain, partly modulated by transporters, which are highly expressed in brain endothelial cells (BECs). Transporters mediate the uptake or efflux of compounds to and from the brain and they can also challenge the delivery of drugs for the treatment of Alzheimer's disease (AD). Currently there is a limited understanding of changes in BBB transporters in AD. To investigate this, we generated brain endothelial-like cells (iBECs) from induced pluripotent stem cells (iPSCs) with familial AD (FAD) *Presenilin 1* (*PSEN1*) mutation and identified AD-specific differences in transporter expression compared to control (ctrl) iBECs. We first characterized the expression levels of 12 BBB transporters in AD-, Ctrl-, and isogenic (*PSEN1* corrected) iBECs to identify any AD specific differences. We then exposed the cells to focused ultrasound (FUS) in the absence (FUS^only^) or presence of microbubbles (MB) (FUS^+MB^), which is a novel therapeutic method that can be used to transiently open the BBB to increase drug delivery into the brain, however its effects on BBB transporter expression are largely unknown. Following FUS^only^ and FUS^+MB^, we investigated whether the expression or activity of key transporters could be modulated. Our findings demonstrate that *PSEN1* mutant FAD (*PSEN1*^*AD*^) possess phenotypical differences compared to control iBECs in BBB transporter expression and function. Additionally, we show that FUS^only^ and FUS^+MB^ can modulate BBB transporter expression and functional activity in iBECs, having potential implications on drug penetration and amyloid clearance. These findings highlight the differential responses of patient cells to FUS treatment, with patient-derived models likely providing an important tool for modelling therapeutic effects of FUS.

## Introduction

Alzheimer's disease (AD) is the most common cause of dementia worldwide, with prevalence and incidence increasing with age [1]. Although most cases of AD develop symptoms after the age of 65 (known as late-onset AD or sporadic AD), approximately 5 ​% of AD patients develop symptoms before the age of 65 and this is described as early onset AD, also known as a type of familial AD (FAD) [2]. FAD is caused by known mutations and cases appear to be inherited in an autosomal dominant manner. Mutations in three genes have been reported to play a role in FAD, including presenilin 1 *(PSEN1),* presenilin 2 *(PSEN2)* and amyloid precursor protein *(APP*) [3].

AD is a neurodegenerative disease which is largely characterised by two neuropathological hallmarks: 1 extracellular accumulation of amyloid-β (Aβ) peptide and 2 intracellular accumulation of hyperphosphorylated tau protein which subsequently forms tau tangles, leading to loss of neurons and neuronal death [4]. In addition, emerging evidence indicates that patients with AD have a dysfunctional blood-brain barrier (BBB) [5,6]. The BBB is a physical, metabolic, and immunological barrier that plays a role in maintaining homeostasis in the brain [7]. Its main roles are to supply nutrients to, and exclude waste products from the brain and regulate the passage of cells and molecules from the systemic circulation to the central nervous system (CNS) to maintain a highly controlled microenvironment for neuronal function. The BBB is primarily composed of brain endothelial cells (BECs), astrocytes, and pericytes, with BBB cells also interacting with neurons and ​microglia to form the neurovascular unit (NVU) [8]. BECs are tightly connected to each other via tight junctions (TJs) and adherens junctions (AJs), including Occludin, Claudin-5, zonula occludens-1 (ZO-1) and VE-Cadherin, respectively [9]. TJs and AJs together control the permeability of the BBB and regulate the passive entry of ions, molecules, and nutrients from the blood to the brain, while protecting the brain from potentially harmful endogenous and exogenous substances, infiltrating immune cells and microorganisms [10]. Drug transport into the brain is also selectively controlled by BECs [4,11,12].

Moreover, the exchange of molecules between the brain and the blood is controlled by BBB transporters expressed in BECs [[12], [13], [14], [15]]. At the BBB multiple different transporter families are expressed. The function of transporters can broadly be divided by pumping molecules “in or out” of the brain, and these are expressed in each of the cellular components of the BBB [16]. BBB transporters include members of ATP-binding cassette (ABC) superfamily and the solute carrier (SLC) superfamily [17,18]. While the ABC transporters, which use the energy from ATP hydrolysis, function mostly as efflux transporters, SLC transporters facilitate passive transport and play a crucial role in transporting small molecules from the blood into the brain [17]. In addition to these mechanisms, specific macromolecules primarily utilize receptor-mediated transcytosis (RMT) as their primary pathway for accessing the brain [19]. RMT entails the interaction of macromolecules, including antibodies and peptides, with specific receptors resulting in the formation of ligand-receptor complexes [20]. Importantly, some BBB transporters are associated with AD and BBB dysfunction. For example, P-glycoprotein (P-gp), which is a member of the ABC transporter superfamily, has been reported to play a major role in the clearance of Aβ from the brain in mouse models [21] and the dysregulation of this efflux transporter might be associated with AD pathogenesis [22]. Moreover, Multidrug resistance protein 1 (MRP1), another member of the ABC transporter family, is expressed in the apical side of BECs and it plays an important role in mediating inflammatory responses and acts as a defence against the accumulation of reactive oxygen species (ROS) in the brain by pumping out toxins (e.g., glutathione complexes), consequently protecting BBB function [23]. In addition, MRP1 plays a crucial role in regulating the efflux of Aβ peptides from the brain to blood [24]. Furthermore, the SLC family is a large group of transport proteins that play a crucial role in moving various substances, such as ions, neurotransmitters, and nutrients, across cell membranes, including the BBB. Various members of the SLC transporter superfamily have been associated with dysfunction of AD and other neurological disorders, including the Solute Carrier Family number 2 Member 1 (*SLC2A1)* also known as Glucose Transporter 1 (GLUT1) [25], the Solute Carrier Family 22 Member 8 (*SLC22A8),* also known as the Organic Anion Transporter 3 (OAT3) [26], the Solute Carrier Organic Anion Transporter Family Member 1A2 *(SLCO1A2*), also known as the Organic Anion-Transporting Polypeptide 1A2 (OATP12) [27,28] and others. The Low-Density Lipoprotein Receptor-Related Protein 1 (LRP1) transporter, member of the low density lipoprotein (LDL) receptors which is transported via RMT, plays a crucial role in the transport of Aβ and post-synaptic receptors [29]. LRP1 is involved in various cellular processes, including the clearance of Aβ, which is known to be dysfunctional in AD [30]. Likewise, the BBB restricts >98 ​% of small drugs and almost 100 ​% of large drugs from entering the brain [31,32], with BBB transporters playing a crucial role in drug restriction [33]. Thus, the BBB and its transporters form a major impediment for researchers developing drugs that target the brain for the treatment of CNS disorders, such as AD [34]. Moreover, dysfunction of the BBB associated with AD likely further hinders drug delivery due to the altered physiological conditions of the BBB and the brain [35], which can lead to ​altered drug metabolism. However, the mechanisms underlying BBB dysfunction in relation to transporter function are yet to be fully investigated in the human [4,17,36].

Focused ultrasound (FUS) together with gas-filled microbubbles (FUS^+MB^) has been shown to temporarily open the BBB and increase therapeutic permeability into the brain in both *in vitro* and *in vivo* studies. [[37], [38], [39], [40], [41], [42]]. This method has been shown to be safe in animals and a limited number of human studies and importantly, it has been shown to aid in drug delivery [42,43] and Aβ clearance in AD mouse models [[44], [45], [46]]. On the other hand, FUS without microbubbles (MBs) (FUS^only^) has been shown to improve memory, cognitive and motor functions and mitigate AD pathogeneses while being safe and efficient as shown in animals and early-stage clinical trials in patients [46,47]. Although the effects of FUS^only^ and FUS^+MB^ have been shown to be promising in modulating the brain microenvironment and drug delivery, respectively, their effects on BBB transporters, particularly in the context of AD and in patient-derived models, have not been investigated.

Since changes in BBB transporter expression and function in AD have not been extensively investigated, we aimed to address this in the present study. Here we utilized a patient-derived FAD iBEC model, which we have previously reported to exhibit characteristics of FAD [39]. We first investigated differences in BBB transporter expression in FAD versus control cells to gain insight into possible dysfunction in BBB transporters in FAD. We then investigated the effects of FUS^only^ and FUS^+MB^ on key BBB transporter expression as well as the activity of two key BBB efflux transporters, P-gp and MRP1. Finally, we investigated the effects of FUS^only^ and FUS^+MB^ on P-gp-mediated Aβ uptake. Our results identified differences in transporter expression and activity between FAD and control iBECs, and we also show that the expression of some transporters is altered following FUS treatments depending on the disease status. Overall, this study provides new insights into BBB transporter changes in FAD and reveals potential modulatory effects of FUS on BBB transporter activity.

## Methods

### Study design

The aim of this research study was to investigate the effects of FAD on BBB transporters and the effect of FUS on transporter expression and function using an *in vitro* BBB model. To investigate this, we used patient-derived iPSCs to derive brain endothelial-like cells. The use of hiPSCs offers significant advantages, including patient-specificity, enabling the investigation of patient differences [48]. In this study a total of six hiPSC lines were used, including two unrelated healthy control lines, two FAD lines harbouring the *PSEN1* exon 9 deletion and two analogous isogenic *PSEN1* exon 9-corrected controls lines [31,40]. iBECs were generated in a controlled laboratory environment and further exposed to FUS treatments, and their responses were investigated in two different timepoints (immediate and 24 ​h timepoint). Additionally, since the effects of the FUS treatments on BBB transporter expression are unknown, we investigated BBB expression and activity using immunofluorescence, RT-qPCR and functional assays following FUS exposure. The samples sizes were selected based on previous experiments performed by our group and based on previous studies in the field [39,42,49,50]. To assess the suitability of iBECs in transporter examination, iBECs were compared for BBB marker and transporter expression to a human immortalized BEC line, hCMEC/D3.

### hiPSC expansion and iBEC differentiation

**Fig. S1:**
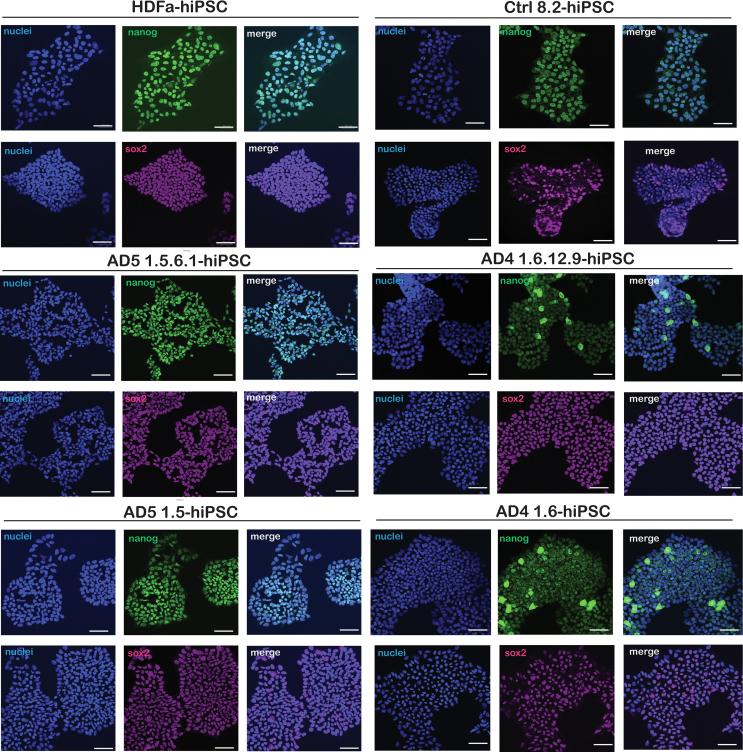
hiPSC characterization for cell lines used in this study. Representative immunofluorescence images for Ctrl-hiPSC, *PSEN1*^cor^-hiPSC and *PSEN1*^AD^-hiPSC of pluripotency markers nanog (green) and sox2 (magenta) (20× magnification -Z stack, Hoechst counterstain). Scale bar, 50 ​μm.

**Fig. S2:**
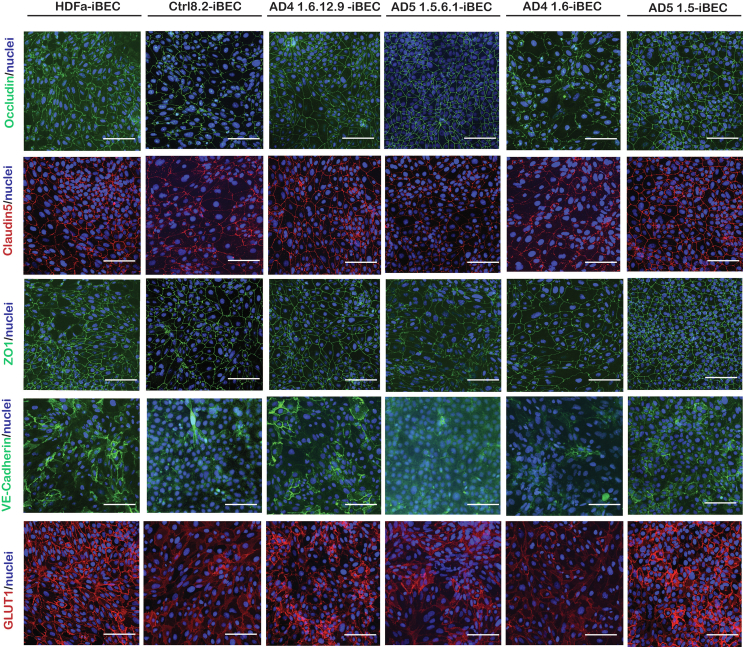
iBEC differentiation characterized by immunofluorescence to confirm tight-adherent junctions and BBB transporter expression in all lines used in this study. Representative immunofluorescence images for Ctrl, *PSEN1*^*COR*^ and *PSEN1*^AD^-iBEC of occludin (green), claudin-5 (red), ZO1 (green), VE-Cadherin (green) and GLUT1(red) (20× magnification -Z stack, Hoechst counterstain). Scale bar, 50 ​μm.

**Fig. S3:**
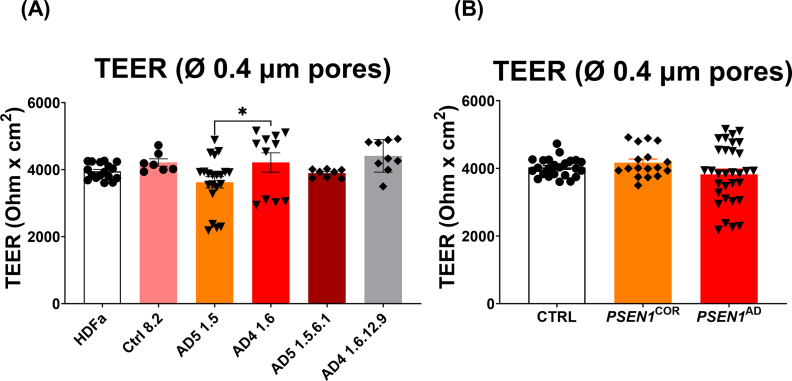
TEER measurement in Ctrl, *PSEN1*^*COR*^ and *PSEN1*^AD^-iBECs to characterize iBEC monolayer integrity. *Trans*-endothelial electrical resistance (TEER, Ohm/cm2) measurement in the six lines used in the study: Ctrl-iBEC (HDFa; Ctrl 8.2), *PSEN1*^*COR*^-iBEC (AD4 1.6.12.9; AD5 1.5.6.1) and *PSEN1*^AD^-iBEC (AD4 1.6; AD5 1.5). N ​= ​2 biological replicates and a minimum of n ​= ​3 for independent replicates per line. Statistical analysis was completed by using one-way ANOVA, error bars ​= ​SEM. ∗P ​< ​0.01.

All hiPSC lines were expanded on human recombinant vitronectin coated culture plates in StemFlex medium (Thermo Fisher), as previously described [31]. All the lines studied were successfully characterised using pluripotency markers nanog and SOX2 (Fig. S1). In addition, all lines exhibited a normal karyotype and tested negative for mycoplasma (Table S1). hiPSCs were induced to differentiate into iBECs as previously described [50,51], and optimised by us [39]. Briefly, high quality hiPSCs were detached and singularized using Accutase (Thermo Fisher) and re-plated at 2.0 ​× ​10^5^ – 2.5 ​× ​10^5^ ​cells/well in a 6-well plate, pre-coated with Matrigel (Corning) in StemFlex ​+ ​10 ​μM Y27632 (ROCKi, Stemcell technologies). After 24 ​h, the media was changed to growth media without ROCKi and daily media changes were performed until day-1 with StemFlex. On day 0, the media was changed to Unconditioned (UM) media, composed of DMEM/F12+Glutamax (1X), Knockout serum replacement (20 %) ​+ ​MEM nonessential amino acids (1x) (all from Thermo Fisher) and β-mercaptoethanol (0.1 ​mM) (Sigma) to start spontaneous differentiation. After this step, media was changed every other day. After 6 days in UM, the media was switched to ECSR1+FGFb+RA, composing of Endothelial serum-free medium (Thermo Fisher) (1x) ​+ ​B27 supplement (2 %) (Thermo Fisher) ​+ ​FGF-2 (20 ​ng/mL) (Peprotech) and Retinoic acid (10 սM) (Sigma). After 2 days in ECSR1+FGFb+RA (day 8 of differentiation) cells were re-plated and purified. To purify the cells, media was removed, and cells were treated with Accutase for approximately 30 ​min at 37 ​°C to detach the cells. Cells were flushed off the plate using D-PBS in order to break up the clumps, assuring single cell suspension. Cells were centrifuged at 300×*g* for 5 ​min and re-suspended into ECSR1+FGFb+RA. Cell count was then performed, and cells re-plated on Collagen IV (400 ​μg/mL, Sigma) ​+ ​Fibronectin (100 ​μg/mL, Thermo Fisher) coated plates at the desired cell densities as previously reported [39]. To confirm the integrity of the iBEC monolayer, *trans*-endothelial electrical resistance (TEER) was measured. For this, cells were cultured in the upper compartments of 0.4 ​μm pore polyester Transwell® 6.5 ​mm inserts (Corning), at a density of 3.0 ​× ​10^5^ ​cells/insert- and TEER was measured using the EVOM3 Volt/Ohm meter. All lines exhibited TEER values of over 2000-Ω x cm^2^ and expressed BEC markers (occluding, claudin-5, ZO-1 and VE-cadherin) via immunofluorescence, confirming iBEC differentiation (Fig. S2 & S3).

### Human immortalized cerebral microvascular endothelial cell (hCMEC/D3) expansion

As a comparison to the iBECs derived from hiPSCs, we also investigated the expression of epithelial, endothelial and BBB markers as well as BBB transporters in hCMEC/D3 cells (Merck Millipore, SCC066), which are human immortalized cerebral microvascular endothelial cells. Briefly, cells were cultured in Endothelial serum-free medium (Thermo Fisher) supplemented with 5 ​% FBS (Life Technologies), 1.4 ​μM hydrocortisone (Sigma), 5 ​μg/mL ascorbic acid (Sigma) and 10 ​mM HEPES (Life Technologies) on T25 ​cell culture flasks (Thermo Fisher Scientific), coated with Collagen IV (400 ​μg/mL, Sigma) ​+ ​Fibronectin (100 ​μg/mL, Thermo Fisher) for 1 ​h at 37 ​°C prior to cell expansion. Medium was changed every 2 days until the plate achieved 80–90 ​% confluence to be replated into a 24 well plate for RNA collection.

### Immunofluorescence for hiPSC and iBEC characterization

Immunofluorescence was used to detect the expression of pluripotency markers in iPSCs and BBB-specific markers and transporters in iBECs. Briefly, cells were rinsed twice with D-PBS for 5 ​min and either fixed with ice-cold methanol for 5 ​min on ice or with 4 ​% paraformaldehyde (PFA) at room temperature (RT) for 20 ​min (refer to Table S2, for antibody-specific information). Following fixation, PFA-fixed cells were permeabilized with 0.3 ​% triton-X for 15 ​min. Cells were then blocked with 2 ​% BSA and 2 ​% normal goat serum (Sigma) in D-PBS for 1 ​h at RT. Primary antibodies were diluted in blocking solution and incubated overnight at 4 ​°C. The following day, cells were washed three times with D-PBS, and incubated with fluorophore-labelled secondary antibodies (goat anti-rabbit Alexa 488 or goat anti-mouse Alexa 594 from Thermo Fisher, all added 1:250) diluted in blocking solution for 1 ​h at RT, protected from light. Cells were then washed three times with D-PBS, followed by treatment with Hoechst counterstaining (1:5000), and coverslips were mounted with ProLong Gold Antifade (Thermo Fisher). Images were obtained using a 20× objective with a Zeiss 780 confocal microscope in Z stack acquisition.

### RNA extraction, cDNA synthesis and quantitative real-time PCR

For gene expression analysis of transporters in iBECs and hCMEC/D3, culture medium was first removed, and cells were rinsed once with D-PBS. Cells were then treated with TRIZOL ™ reagent (Thermo Fisher) and scraped off the culture plate using a pipette tip. RNA was extracted using the RNA Miniprep kit (Zymo Research) according to the manufacturer's instructions. After that, the quality and quantity of RNA was measured using a Nanodrop Spectrophotometer and RNA was converted to cDNA using SensiFAST™ cDNA synthesis kit (Bioline). *18S* was used as a housekeeping gene to normalize qRT-PCR results. Following the manufacturer's instructions, cDNA samples were combined with SensiFAST™ SYBR® Lo-ROX (Bioline) and gene-specific primers to form the reaction solution. The qRT-PCR run was performed in triplicate for each sample on QuantStudio™ 5 Real-Time PCR system (Thermo Fisher Scientific). Briefly, the samples were run for 2 ​min at 95 ​°C followed by 40 cycles of 5 ​s at 95 ​°C then 30 ​s at 60 ​°C. The CT values of each gene was normalized to CT values of *18S* (ΔCT values). ΔΔCt values were then calculated as 2^(−ΔCt)^ and multiplied by 10^6^. Multiplied ΔΔCt values were log-transformed prior to graphical presentation. Primer sequences from the genes used in this study are presented in supplementary material (Table S3).

### Focused ultrasound treatment

The effects of focused ultrasound (FUS) with or without microbubbles (MBs) were tested on iBEC transporter expression and function. The experiments were performed 48 ​h following iBEC purification on collagen IV and fibronectin coating, as previously described by us [39]. For immunofluorescence, transporter functional activity and RNA sample collection assays, cells were grown on 24-well plates. For TEER measurement assays cells were grown on Ø 0.4 ​μm pore Transwell inserts. Cells were tested under three different conditions 1 untreated (UT), 2 focused ultrasound alone (FUS^only^), and 3 focused ultrasound with microbubbles (FUS^+MB^). MBs were prepared in-house as previously described [44,52] and further optimised by our group to use in *vitro* patient-derived model [42]. MBs were added at 20 ​μL per well in 24-well plates and 10 ​μL per well in Transwell inserts as previously optimised by us [39]. Finally, cells were exposed to FUS (Sonic Concepts) using 0.3 ​MPa peak rarefactional pressure, 50 cycles/burst, burst period 20 ​ms, 286 ​kHz center frequency, and a 120 ​s sonication time as previously described by us [39]. For the FUS^only^ condition, cells were sonicated following the same parameters without MB addition to the wells. Following treatment, cells were analyzed at two time-points: within 1 ​h after treatment (immediate timepoint) and at 24 ​h after treatment, to capture iBEC barrier opening and closing, respectively, as previously demonstrated by us and others [39,53,54].

### Rhodamine accumulation assay to measure P-gp activity

The functional activity of P-gp in iBEC monolayers was measured by the intracellular accumulation of fluorescent rhodamine 123, which is a substrate for P-gp. The P-gp activity assay using rhodamine 123 was performed as previously described [39,55,56]. Briefly, 48 ​h following purification on collagen IV and fibronectin, iBECs were first exposed to FUS^only^ or FUS^+MB^ or left untreated and rhodamine 123 uptake was performed immediately (Imm) or 24 ​h following treatment. For this, cells were first pre-treated with 10 ​μM of the P-gp inhibitor Cyclosporin-A (CsA, Sigma) or left untreated (only treated with Hanks' balanced salt solution (HBSS)) and incubated at 37 ​°C for 30 ​min. Cells were then incubated with 10 ​μM rhodamine 123 with or without CsA in HBSS at 37 ​°C for 2 ​h. Following incubation, cells were washed with ice-cold D-PBS for 5 ​min and then lysed with RIPA cell lysis buffer (Sigma) for 10 ​min on a microplate shaker. Fluorescence intensity of cell lysates was measured on a plate reader (Biotek Synergy H4 Multi Mode Plate Reader) using the following wavelengths: 485 ​nm excitation and 530 ​nm emission. Results were normalized to total protein amount, which was quantitated using a Pierce ™ BCA protein assay (Thermo Fisher Scientific), according to the manufacturer's instructions.

In the absence of CsA, rhodamine 123 is expelled from the cells via the efflux function of P-gp, however, when P-gp is inhibited by CsA, intracellular accumulation of rhodamine 123 occurs. Thus, elevated intracellular accumulation of rhodamine 123 following P-gp inhibition by CsA, equates to lower activity of P-gp. To confirm active P-gp function, the fold change of rhodamine 123 uptake in +CsA treated cells to non-CsA treated cells for each group (-CsA vs ​+ ​CsA) was calculated making sure that the accumulation of rhodamine was at least 30 % higher in +CsA cells compared to -CsA cells in the UT conditions [55] (Fig. S8A). Following confirmation of P-gp activity, the effects of FUS^only^ and/or FUS^+MB^ treatment on rhodamine 123 accumulation (P-gp activity) was then measured by calculating fold change in rhodamine 123 accumulation compared to UT in +CsA treated cells at the immediate and 24 ​h timepoints.

### Calcein AM accumulation assay to measure MRP1 activity

The functional activity of MRP1 in iBEC monolayers was measured by the intracellular accumulation of fluorescent calcein-AM, which is a substrate for MRP1. The MRP1 activity assay using calcein AM was performed as previously described [55,56]. Briefly, 48 ​h following purification on collagen IV and fibronectin, cells were first exposed to FUS^only^ or FUS^+MB^ or left untreated and calcein-AM uptake was performed immediately (Imm) or 24 ​h following treatment. For this, cells were first pre-treated with 12.5 ​nM MK-571 (Sigma), which is an MRP1 inhibitor, or left untreated (treated only with DMEM/F12) and then incubated at 37 ​°C for 30 ​min. Cells were then treated with or without MK-571 along with 5 ​nM calcein-AM (Sigma) diluted in DMEM/F12 for 1 ​h at 37 ​°C. Following incubation, cells were washed with ice-cold D-PBS for 5 ​min and then lysed with RIPA cell lysate buffer (Sigma) for 10 ​min on a microplate shaker. Fluorescence intensity of cell lysates was measured on a plate reader (Biotek Synergy H4 Multi Mode Plate Reader), with the following wavelengths: 496 ​nm excitation and 560 ​nm emission. First, the fluorescence values were normalized to total protein obtained by BCA assay (as previously described by Stebbins et al. [55]. Then, the fold change of +MK-571 treated samples to non-MK-571 samples was analyzed using the UT Imm samples ensuring that the accumulation of calcein-AM was in a range of (37 ​%–100 ​%) (Fig. S8B), indicating the presence of MRP1 activity, as previously reported [57]. After this, cells that had been exposed to MK-571 were used to examine the effects of FUS^only^ and FUS^+MB^ treatment on calcein AM accumulation at the immediate and 24 ​h timepoints.

### Analysis of P-gp-mediated intracellular amyloid-Aβ accumulation

To measure P-gp-mediated Aβ clearance, the intracellular accumulation of FITC-conjugated Aβ_42_, an isoform of Aβ known to accumulate and contribute to the formation of Aβ plaques in the brain [58,59], was assessed following iBEC exposure to the P-gp inhibitor CsA. Briefly, 48 ​h following purification on collagen IV and fibronectin, iBECs were exposed to FUS^only^ or FUS^+MB^ or left untreated. Then immediately or 24 ​h following treatment, cells were incubated with 10 ​μM CsA (Sigma) or without P-gp inhibitor (HBSS only) for 1 ​h at 37 ​°C. Following CsA incubation, cells were then incubated with 5 ​μM of FITC-conjugated Aβ_42_ (Bachem) diluted in HBSS for 1 ​h 30 ​min at 37 ​°C. After this, cells were washed with ice-cold D-PBS for 5 ​min and then lysed with RIPA cell lysate buffer (Sigma) for 10 ​min on a microplate shaker. Fluorescence intensity of cell lysates was measured on a plate reader (Biotek Synergy H4 Multi Mode Plate Reader), as follows: 475 ​nm excitation and 650 ​nm emission for Aβ assay. As with the rhodamine 123 and calcein-AM uptake assays, fluorescence values of Aβ accumulation were first normalized to total protein and then effects of FUS^only^ and FUS^+MB^ compared to UT on Aβ accumulation within +CsA treated cells was analyzed as described above (Fig. S8(G-I)).

In the absence of CsA, Aβ is expelled from the cells via the efflux function of P-gp, however, when P-gp is inhibited by CsA, intracellular accumulation of Aβ occurs. Thus, elevated intracellular accumulation of Aβ following P-gp inhibition by CsA, equates to lower levels of P-gp-mediated Aβ clearance. To confirm active P-gp function, the fold change of Aβ in +CsA treated cells to non-CsA treated cells for each group (-CsA vs ​+ ​CsA). Following confirmation of P-gp activity, the effects of FUS^only^ and/or FUS^+MB^ treatment on Aβ intracellular accumulation (P-gp activity) was then measured by calculating fold change in Aβ intracellular accumulation compared to UT in +CsA treated cells at the immediate and 24 ​h timepoints.

### Statistical analysis

Statistical analysis was performed using GraphPad Prism version 9.4.0. Data were analyzed using Student t-test and one-way ANOVA and the specific multiple comparison tests are described in each figure legend. Welch's correction was used if samples exhibited unequal standard deviation (SD). Normality of values was confirmed with a QQ plot. Statistical significance was defined as *P* ​< ​0.05. Error bars are shown as standard deviation (SD) or SEM. The number (N) ​= ​biological replicates (for hiPSCs and iBECs) and (n) ​= ​independent replicates used for each experiment are defined in each figure legend.

## Results

### Comparison of expression of BBB, endothelial and epithelial markers in iBECs and hCMEC/D3 cells

**Fig. S4:**
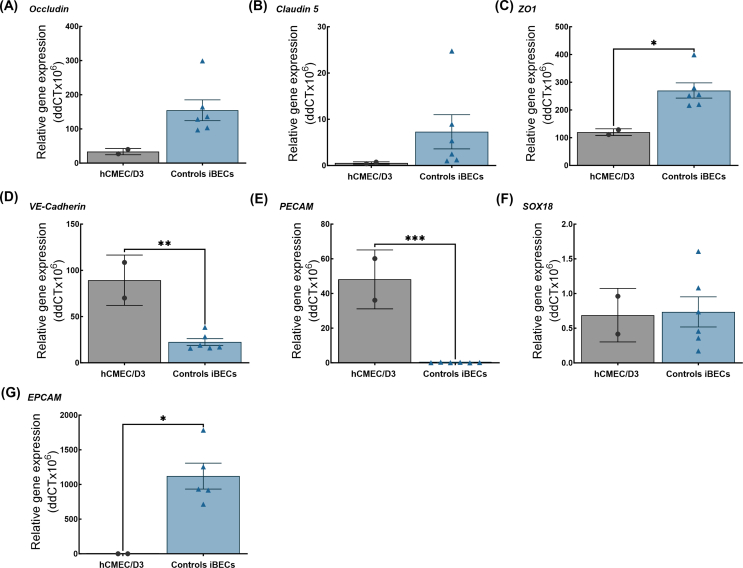
Expression of endothelial and epithelial markers in hCMEC/D3 and Ctrl-iBECs. (A to H) Relative gene expression to housekeeping gene *18S* of endothelial and epitelial markers, including *Occludin, Claudin – 5, ZO1, VE-Cadherin, PECAM, SOX18, TAL1* and *EPCAM* in hCMEC/D3 and iBECs shown as averaged ΔCtx10^6^. N ​= ​1 (hCMEC/D3) and N ​= ​2 (Ctrl-iBECs) for biological replicates and a minimum of n ​= ​2–3 for independent replicates per line. Statistical analysis was completed by using Student's test, error bars ​= ​SEM. ∗P ​< ​0.05, ∗∗P ​< ​0.01 and ∗∗∗P ​< ​0.001.

To validate the iBEC model for studying BBB transporter changes in AD, we first conducted a comparison between our iBECs and a human immortalized cerebral microvascular endothelial cell line, hCMEC/D3, for similarities and differences in BBB, endothelial and epithelial markers. Interestingly, no significant differences were found in the relative expression of BBB markers *Occludin* and *Claudin-5* or the endothelial transcription factor *SOX18* between iBECs and hCMEC/D3 cells (Fig. S4A-B & F). In contrast, hCMEC/D3 cells demonstrated significantly higher levels of *VE-Cadherin* and *PECAM* expression, while the relative expression of *ZO1* and *EPCAM* was significantly higher in iBECs (Fig. S4). These results are in line with previous reports of iBECs being a mixture of endothelial and epithelial cells [60], which importantly exhibit a high level of BBB marker expression.

### Comparison of BBB transporter expression in control and *PSEN1*^AD^ iBECs reveals *PSEN1* and *non-PSEN1* related changes.

As the role of BBB transporters in AD is poorly understood, we first compared the mRNA levels of selected BBB transporters between iBECs containing *PSEN1* FAD mutation (*PSEN1*-AD), isogenic *PSEN1*-corrected (*PSEN1*^COR^) and unrelated healthy controls (Ctrl). These included 12 highly expressed BBB transporters. The selected BBB transporters investigated in this study included both ABC and SLC superfamily transporters as well as the low-density lipoprotein receptor-related protein-1 (*LRP1)* which is part of the RMT transporter family. All selected transporters used in this study are highly expressed in the brain, and some are also associated with BBB function, dysfunction and/or AD pathophysiology (summarized in Table 1).

**Table 1:** Cell location, function and association with AD of BBB transporters.

TRANSPORTERS	LOCATION	FUNCTION & REF.
*ABCA1*/Cholesterol Transporter	Extensively expressed in brain tissues	Regulates the efflux of cholesterol and phospholipids to APOE's lipidation, recent *in vitro* studies in human model of the BBB demonstrated that the dysregulation of cholesterol affects Aβ exchange [61]. Downregulates the influx of Aβ across the BBB [62].
*ABCB1*/P – Glycoprotein (Pgp)	Expressed in BECs, pericytes, astrocytes and neurons	Downregulated at the BBB during normal aging process [63]. Role in the clearance of Aβ from the brain into blood. Transports xenobiotics across the BBB [17]
*ABCC1*/Multidrug Resistance Protein 1 (MRP1)	Expressed in BECs, astrocytes and pericytes	Low levels of ABCC1 may increase the levels of Aβ40 and Aβ42 in the brain of transgenic mice [64].
*ABCC2*/Multidrug Resistance Protein 2 (MRP2)	Expressed in BECs.	Upregulated in Alzheimer's models (human and animal) [36].
*ABCG2*/Breast Cancer Resistant Protein (BCRP)	Overexpressed in BECs	Major role in mediating the efflux of Aβ in BECs [65]
*ABCG4*/ATP binding cassette subfamily G member 4	Expressed in glial cells, neurons and BECs.	Regulates the efflux of cholesterol [66]. Altered *ABCG4* leads to increases in Aβ secretion [67].
*SLC2A*1/GLUT1 ​= ​Glucose Transporter 1	Expressed in BECs, neurons, astrocytes, and microglia	Role in glucose homeostasis, downregulation of this transporter accelerates BBB disruption, via tight junctions' protein degradation [68].
*SLC22A3*/OCT3- Solute Carrier Family 22 Member 3	Highly expressed in BECs	Role in the uptake of organic anions/cations into the brain [69,70].
*SLC22A8*/OAT3 – Solute Carrier Family 22 Member	Expressed in the brain	Role in the efflux transport (brain to blood) of therapeutic agents (e.g., antivirals and antibiotics) [71]. It has found to be dysregulated in AD brains and preclinical models of AD, which suggest a role in AD pathogenesis [72]
*SLC7A5*/LAT1 – L-type amino acid transporter 1	Highly expressed in the brain	Role in the uptake of essential amino acids and drugs into the BBB [73,74].
*SLCO1A2/*OATP12/Organic anion transporting polypeptide.	Highly expressed in the brains and brain regions	The most important SLCO in human brain due to its high expression levels in the brain. Additionally, it plays a role in the uptake of drugs. Alterations in this gene increase the risk of AD, and it has been associated with cortical Aβ deposition in AD [28]
LRP1 – low-density lipoprotein receptor- related protein-1	Extensively expressed in brain tissues	Plays a major role in regulating Aβ levels in the brain, LRP1 expression is reduced with age and in AD patients [75]. LRP1 has been shown to play a role in controlling the levels of tau in the brain, and downregulation of LRP1 in *vivo* sanimal models of AD, was shown to reduce the tau spread in the brain [76].

**Fig. S5:**
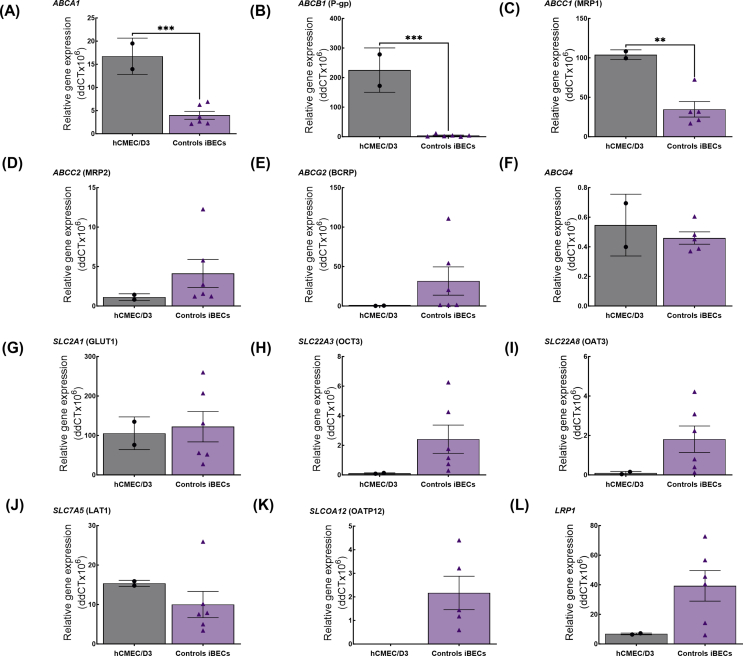
Comparison of relative gene expression for BBB transporters in hCMEC/D3 to Ctrl-iBECs. (A to F) Relative gene expression to housekeeping gene *18S* of ATP-dependent transporters *ABCA1, ABCB1, ABCC1, ABCC2, ABCG2* and *ABCG4* (G to K) solute carrier (SLC) transporters *SLC2A1, SLC22A3* and *SLC7A5* and the (L) *LRP1* in hCMEC/D3 and Ctrl-iBECs shown as averaged of ΔCtx10^6^. N ​= ​1 (Ctrl-hCMEC/D3) and N ​= ​2 (Ctrl-iBECs) for biological replicates and a minimum of n ​= ​3 for independent replicates per line. Statistical analysis was completed by using Student's test, error bars ​= ​SEM. ∗P ​< ​0.05, ∗∗P ​< ​0.01, ∗∗∗P ​< ​0.001, ∗∗∗∗P ​< ​0.0001.

To ensure the presence of the selected transporters in iBECs, we first compared their expression levels between iBECs and hCMEC/D3 cells. The results revealed that out of the 12 BBB transporters analyzed, three ABC transporters (*ABCA1*, *ABCB1* (P-gp) and *ABCC1* (MRP1)) were more highly expressed in hCMEC/D3 cells compared to iBECs (Fig. S5). For the remaining nine BBB transporters analyzed, no significant differences in relative gene expression were observed (Fig. S5). These results indicated a mostly similar level of transporter expression between iBECs and human endogenous BECs, suggesting the feasibility of iBECs for transporter modelling.

**Fig. 1:**
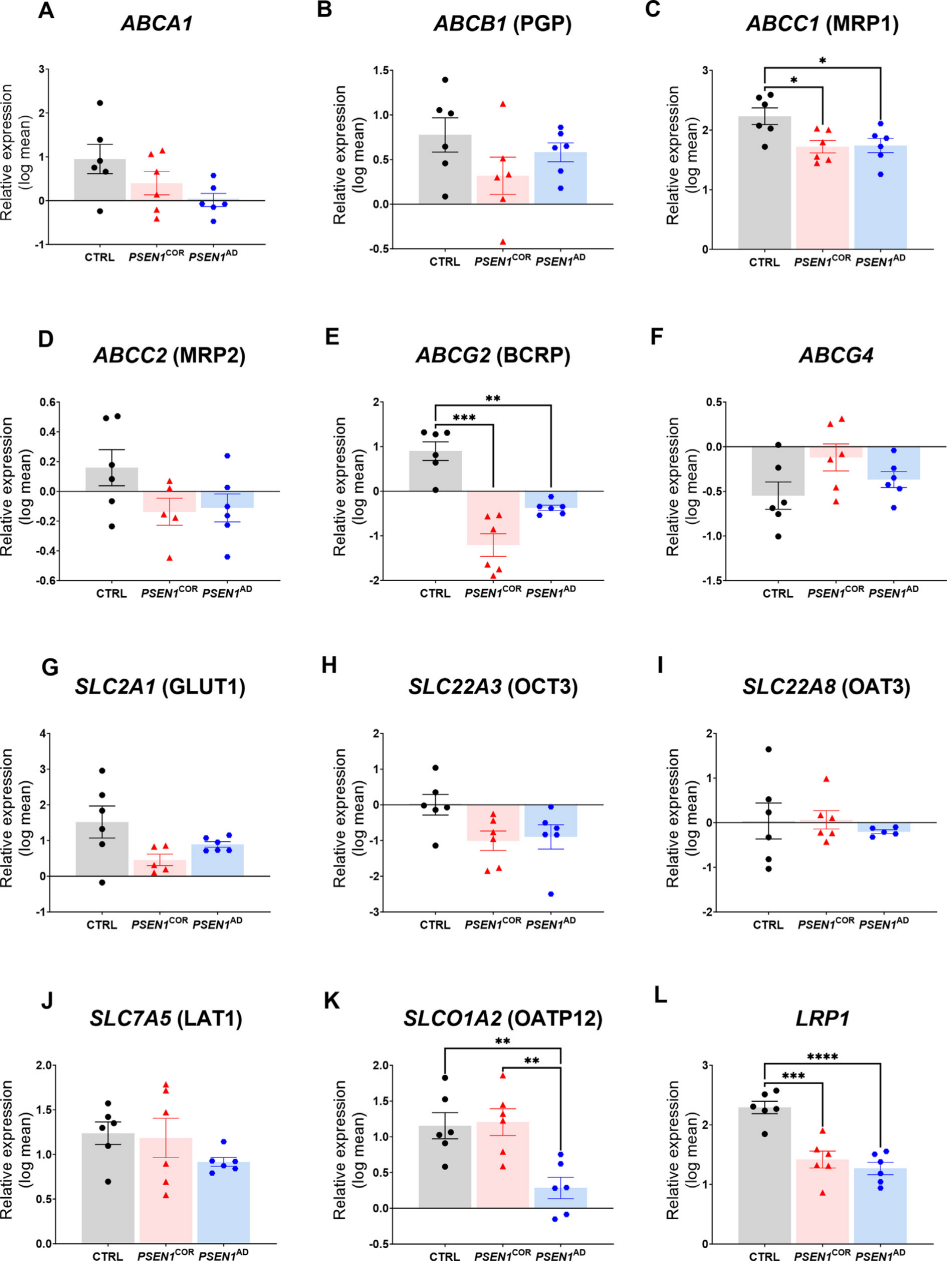
**Expression of BBB transporters in Ctrl-, *PSEN1*^*COR*^- and *PSEN1*^AD^-iBECs** (A to F) Relative gene expression to housekeeping gene *18S* of ATP-dependent transporters *ABCA1, ABCB1, ABCC1, ABCC2, ABCG2* and *ABCG4* (G to K) solute carrier (SLC) transporters *SLC2A1, SLC22A3, SLC22A8, SLC7A5* and *SLCO1A2* and (L) *LRP1* in Ctrl-, *PSEN1*^*COR*^- and *PSEN1*^AD^-iBECs. N ​= ​2 biological replicates and a minimum of n ​= ​3 for independent replicates per line. Statistical analysis was performed by one-way-ANOVA, error bars ​= ​SD. ∗*P* ​< ​0.05, ∗∗*P* ​< ​0.01, ∗∗∗*P* ​< ​0.001, ∗∗∗∗*P* ​< ​0.0001.

Next, Ctrl-, *PSEN1*^*COR*−^ and *PSEN1*^AD^-iBEC lines were compared for differences in the expression of the selected transporters. We found no significant differences in the expression of *ABCA1*, *ABCB1* (PGP) *ABCC2* (MRP2), *ABCG4*, *SLC2A1* (GLUT1), *SLC22A3* (OCT3), and *SLC22A3* (OCT3) and *SLC7A5* (LAT1) between the iBEC groups, however, interestingly, significant differences in expression were identified in the other examined transporter genes (Fig. 1). Of the ATP-dependent transporters both *ABCC1* (MRP1) (Fig. 1C) and *ABCG2* (BCRP) (Fig. 1E) were significantly downregulated in *PSEN1*^*COR*^-iBECs and *PSEN1*^AD^-iBECs compared to Ctrl-iBECs. When the SLC transporters were examined, *SLCO1A2* (OATP12) was significantly downregulated in *PSEN1*^AD^-IBECs compared to Ctrl-iBECs (Fig. 1K). From RMT transporters, we analyzed the levels of *LRP1* which was downregulated in both *PSEN1*^*COR*^-iBECs and *PSEN1*^AD^-iBECs when compared to Ctrl-iBECs (Fig.1L).

Altogether, the results from the initial screening further highlight that some BBB transporters are dysregulated in *PSEN1* mutant individuals, suggesting their potential involvement in FAD pathogeneses. The findings are summarized in Table S4.

### Effects of FUS treatment on gene expression of ABC, SLC and RMT BBB transporters in control and *PSEN1*^AD^ iBECs

FUS^+MB^ is a relatively new technology that can transiently and safely open the BBB to enable drug delivery in *vivo* and *in vitro* studies [39,46]. In addition, FUS^only^ without MBs has been shown to have potentially promising effects on brain cell function [[77], [78], [79]]. However, whether FUS^only^ or FUS^+MB^ elicit any modulatory effects on BBB transporters has not been previously investigated. Thus, to assess the effects of FUS^only^ or FUS^+MB^ treatments on BBB drug transporter expression, we next analyzed the gene expression of the studied transporters in Ctrl-, *PSEN1*^COR^ and *PSEN1*^AD^-iBECs at two different timepoints (immediately and at 24 ​h) following FUS^only^ and FUS^+MB^; and compared the results to UT samples [39].

**Fig. 2:**
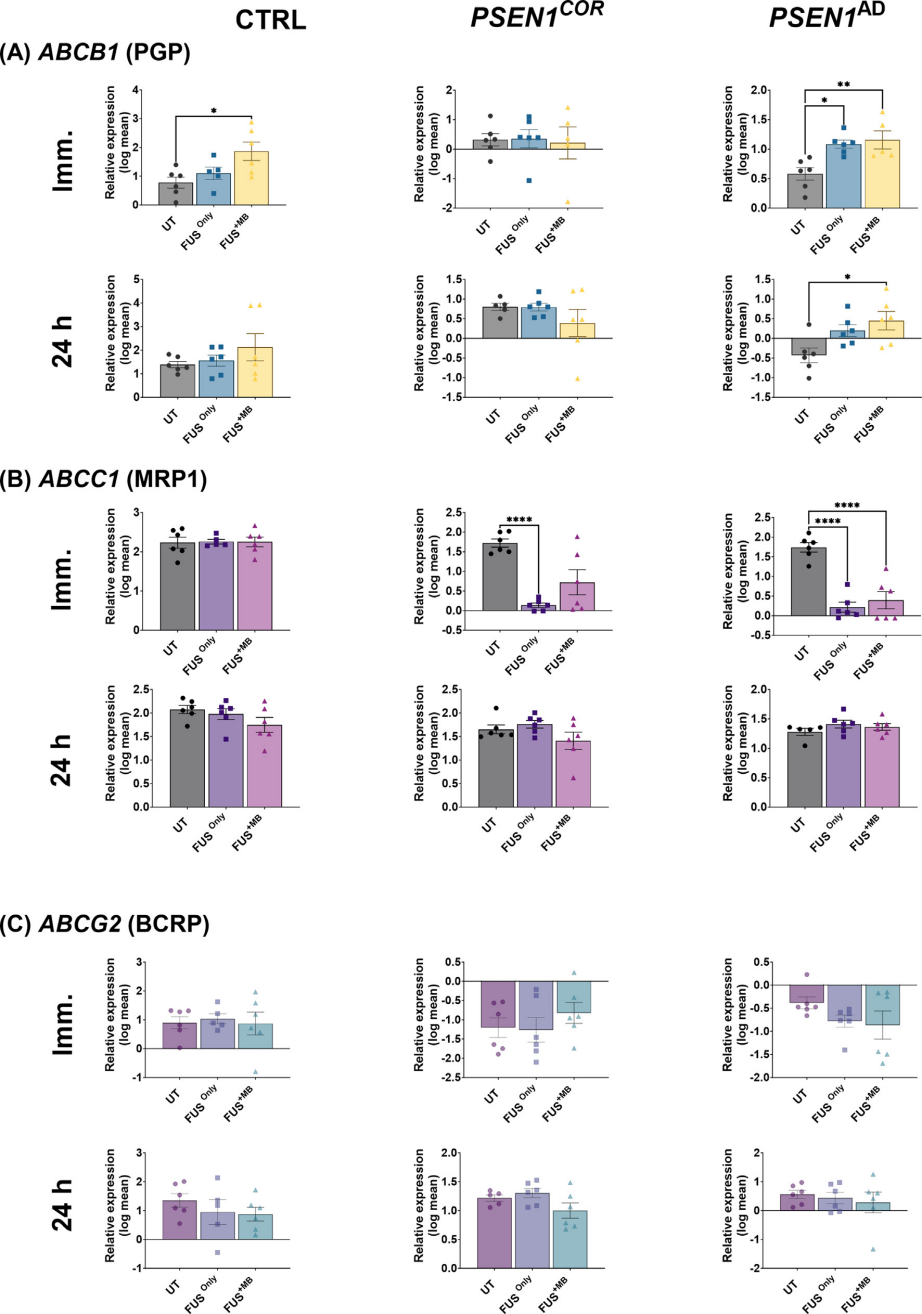
**Relative gene expression of ABC transporters in Ctrl, *PSEN1*^*COR*^-iBEC and *PSEN1*^AD^-iBECs, immediately and 24 ​h after FUS^only^ or FUS^+MB^**. Relative gene expression to *18S* of (A) *ABCB1* (P-gp) immediately and 24 ​h following FUS^only^ or FUS^+MB^ treatments compared to UT for Ctrl-iBEC, *PSEN1*^*COR-*^iBECs and *PSEN1*^AD^-iBECs, (B) *ABCC1* (MRP1) immediately and 24 ​h in FUS^only^ or FUS^+MB^ treatments compared to UT for Ctrl-iBEC, *PSEN1*^cor^-iBEC and *PSEN1*^AD^-iBECs and (C) *ABCG2* (BCRP) immediately and 24 ​h in FUS^only^ or FUS^+MB^ treatments compared to UT for Ctrl-iBEC, *PSEN1*^cor^-iBEC and *PSEN1*^AD^-iBECs. N ​= ​2 biological replicates and a minimum of n ​= ​3 for independent replicates per line. Statistical analysis was completed by using one-way-ANOVA, error bars ​= ​SD. ∗*P* ​< ​0.05, ∗∗P ​< ​0.01, ∗∗∗P ​< ​0.001 and ∗∗∗∗P ​< ​0.0001.

Following FUS treatments, the results revealed that the relative expression of *ABCB1* (P-gp) expression was significantly higher in Ctr-iBECs immediately after FUS^+MB^ treatment when compared to UT samples (Fig. 2A), however no changes were identified after 24 ​h (Fig. 2A). In *PSEN1*^cor^-iBECs neither FUS treatment elicited significant changes in *ABCB1* expression at either time-point (Fig. 2A). In *PSEN1*^AD^-iBECs, the expression of *ABCB1* was, similar to Ctrl-iBECs, significantly increased immediately after FUS^only^ and FUS^+MB^ treatments compared to UT (Fig. 2A), and this increase was also observed 24 ​h after FUS^+MB^ treatment (Fig. 2A).

The relative expression of *ABCC1* (MRP1) was not altered in Ctrl-iBECs immediately and 24 ​h following FUS^only^ or FUS^+MB^ treatments when compared to UT (Fig. 2B). *PSEN1*^cor^-iBECs and *PSEN1*^AD^-iBECs responded similarly to FUS^only^ with the expression of *ABCC1* significantly downregulated, however after FUS^+MB^ treatment *ABCC1* downregulation was only seen in *PSEN1*^AD^-iBECs (Fig. 2B). In all cell groups, no significant changes were observed in *ABCC1* levels 24 ​h following FUS^only^ and FUS^+MB^ treatment when compared to UT (Fig. 2B).

The relative gene expression of *ABCG2* (BCRP) was not altered in any of the cell groups studied (Ctrl-iBECs, *PSEN1*^COR^-iBECs, and *PSEN1*^AD-^iBECs) following FUS^only^ and FUS^+MB^ when compared to UT (Fig. 2C).

**Fig. S6:**
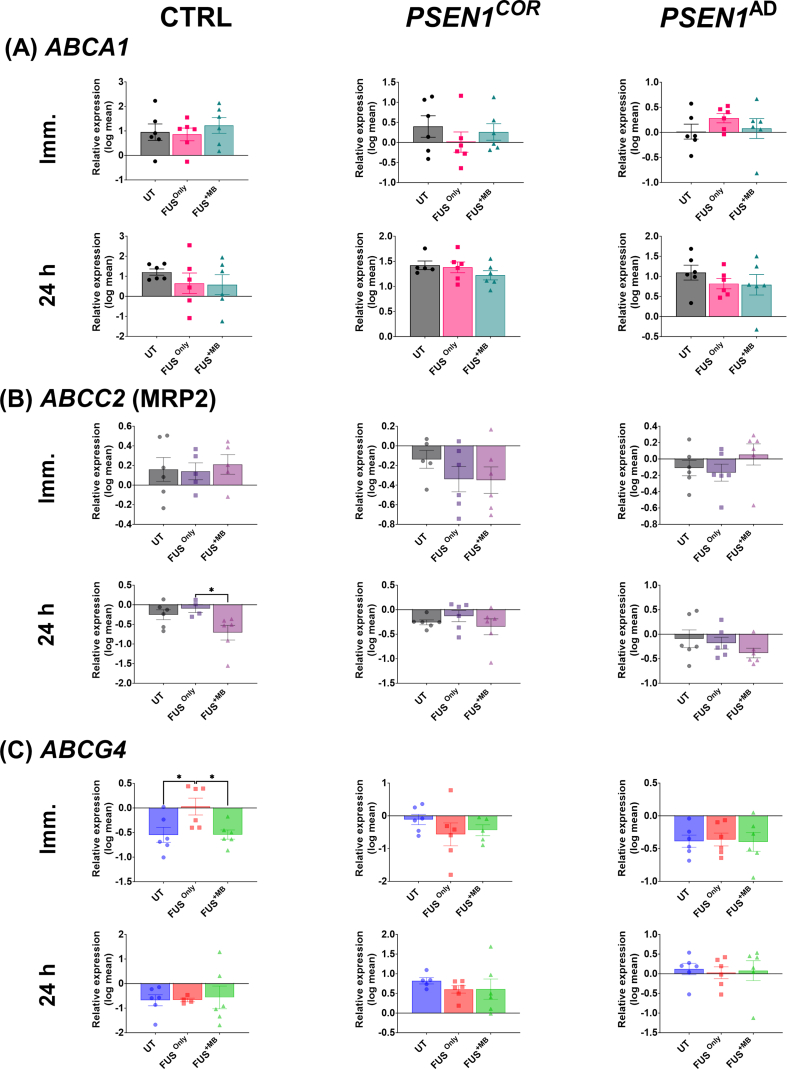
Relative gene expression of ABC transporters in Ctrl, *PSEN1*^*COR*^-iBEC and *PSEN1*^AD^-iBECs, immediately and 24 ​h after FUS^only^ or FUS^+MB^. (A) Relative gene expression to *18S* of *ABCA1* immediately and 24 ​h following FUS^only^ or FUS^+MB^ treatments compared to UT for Ctrl-iBEC, *PSEN1*^*COR-*^iBECs and *PSEN1*^AD^-iBECs. (B) Relative gene expression to *18S* of *ABCC2* (MRP2) immediately and 24h in FUS^only^ or FUS^+MB^ treatments compared to UT for Ctrl-iBEC, *PSEN1*^cor^-iBEC and *PSEN1*^AD^-iBECs.(C) Relative gene expression to *18S* of *ABCG4* immediately and 24h in FUS^only^ or FUS^+MB^ treatments compared to UT for Ctrl-iBEC, *PSEN1*^cor^-iBEC and *PSEN1*^AD^-iBECs N ​= ​2 biological replicates and a minimum of n ​= ​3 for independent replicates per line. Statistical analysis was completed by using one-way-ANOVA, error bars ​= ​SD.∗P ​< ​0.05.

Only minimal changes following FUS treatments were seen in the remaining studied ABC transporters (Fig. S6). *ABCA1* and *ABCC2* (MRP2) were not altered in any of the cell groups following FUS treatments compared to UT (Fig. S6). The relative expression of *ABCG4* was increased immediately after FUS^only^ when compared to UT and FUS^+MB^ (Fig. S6C). Overall these results suggest differences between ABC transporters to FUS modulation with potential patient-specific effects.

**Fig. 3:**
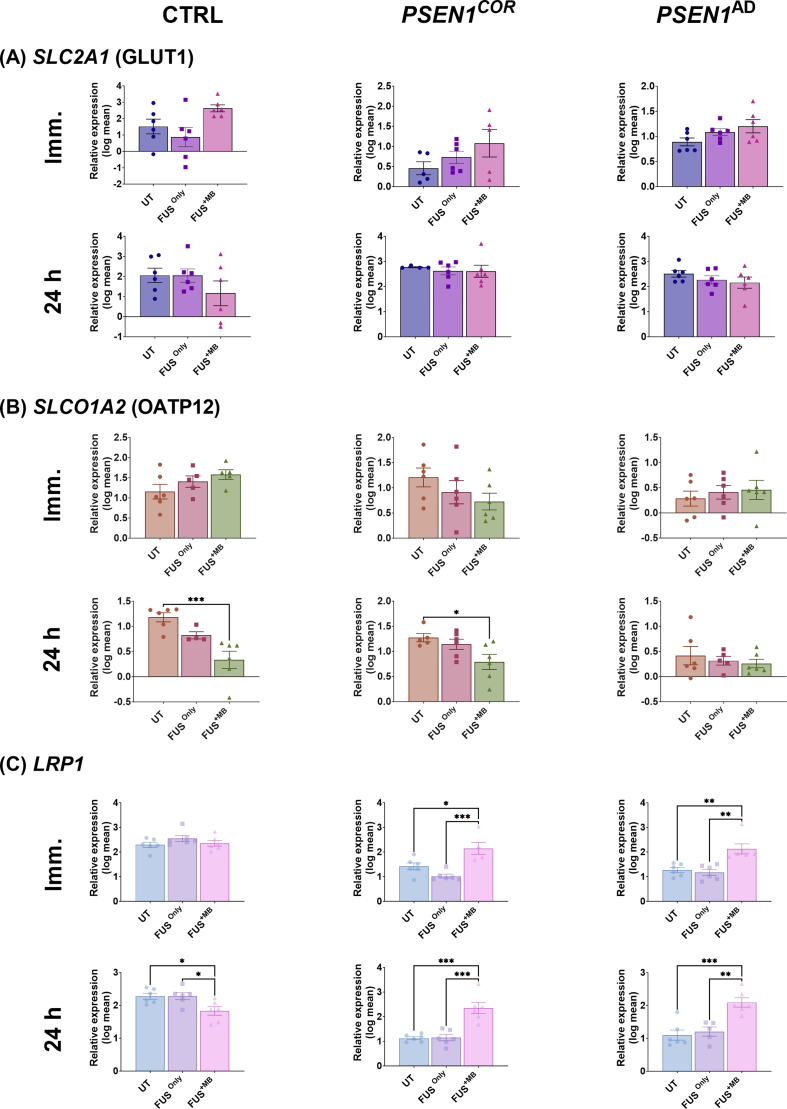
**Relative gene expression for SLC and RMT transporters in Ctrl-, *PSEN1*^*COR*-^iBEC and *PSEN1*^AD^-iBECs, immediately and 24 ​h after FUS^only^ or FUS^+MB^**. Relative gene expression to *18S* of (A) *SLC2A1* (GLUT1) immediately and 24 ​h following FUS ^only^ or FUS^+MB^ treatments compared to UT for Ctrl-iBEC, *PSEN1*^*COR*^-iBECs and *PSEN1*^AD^-iBECs, (B) *SLCO1A2* (OATP12) immediately and 24 ​h following FUS^only^ or FUS^+MB^ treatments compared to UT for Ctrl-iBEC, *PSEN1*^*COR*^-iBECs and *PSEN1*^AD^-iBECs and (C) *LRP1* immediately and 24 ​h following FUS^only^ or FUS^+MB^ treatments compared to UT for Ctrl-iBEC, *PSEN1*^*COR*^-iBECs and *PSEN1*^AD^-iBECs. N = 2 biological replicates and a minimum of n ​= ​3 for independent replicates per line. Statistical analysis was completed by using one-way-ANOVA, error bars ​= ​SD.∗*P* ​< ​0.05, ∗∗*P* ​< ​0.01, ∗∗∗*P* ​< ​0.001 and ∗∗∗∗*P* ​< ​0.0001.

For SLC transporters, the gene expression of *SLC2A1* (GLUT1) was not significantly altered immediately and 24 ​h following FUS^only^ and FUS^+MB^ compared to UT in all cell lines analyzed (Ctrl-iBECs, *PSEN1*^*COR*^-iBECs and *PSEN1*^AD^-iBECs) (Fig. 3A).

Interestingly, the expression of *SLCO1A2* (OATP12), was significantly downregulated in expression when compared to UT in both Ctrl-iBECs and *PSEN1*^COR^ -iBECs 24 ​h following FUS^+MB^, an effect not seen in *PSEN1*^AD^-iBECs (Fig. 3B). In addition, the results from the relative expression of *LRP1* revealed no significant changes in Ctrl-iBECs immediately following FUS^only^ and FUS^+MB^ treatments (Fig. 3C). However, *LRP1* expression was significantly upregulated in *PSEN1*^COR^-iBECs and *PSEN1*^AD^-iBECs immediately and 24 ​h following FUS^+MB^ treatment compared to UT and FUS^only^ conditions (Fig. 3A). In contrast to *PSEN1*^COR^-iBECs and *PSEN1*^AD^-iBECs, Ctrl-iBECs demonstrated significant downregulation of *LRP1* expression 24 ​h following FUS^+MB^ treatment compared to UT and FUS^only^ conditions (Fig. 3C). The findings are summarized in Table S5.

**Fig. S7:**
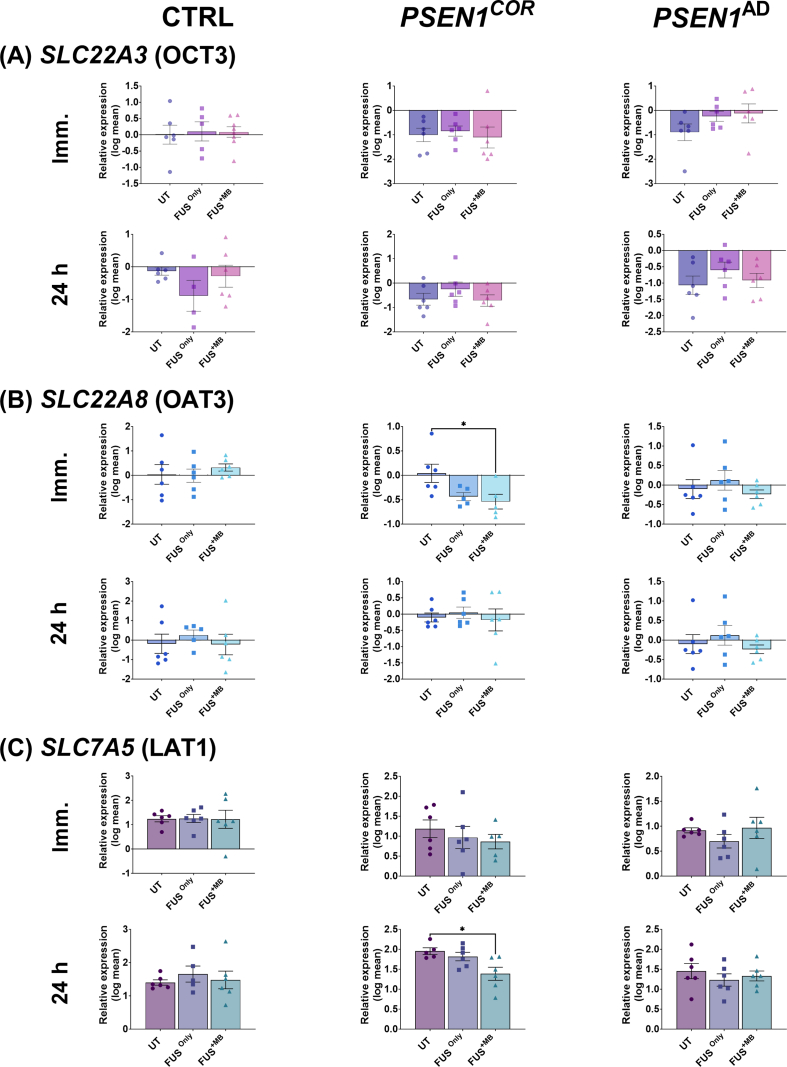
Relative gene expression for SLC transporters in Ctrl-, *PSEN1*^*COR*-^iBEC and *PSEN1*^AD^-iBECs, immediately and 24 ​h after FUS^only^ or FUS^+MB^. (A) Relative gene expression to *18S* of *SLC22A3* (OCT3) immediately and 24 ​h following FUS ^only^ or FUS^+MB^ treatments compared to UT for Ctrl-iBEC, *PSEN1*^*COR*^-iBECs and *PSEN1*^AD^-iBECs. (B) Relative gene expression to *18S* of *SLC22A8* (OAT3) immediately and 24 ​h following FUS^only^ or FUS^+MB^ treatments compared to UT for Ctrl-iBEC, *PSEN1*^*COR*^-iBECs and *PSEN1*^AD^-iBECs. (C) Relative gene expression to *18S* of *SLC7A5* (LAT1) immediately and 24 ​h following FUS^only^ or FUS^+MB^ treatments compared to UT for Ctrl-iBEC, *PSEN1*^*COR*^-iBECs and *PSEN1*^AD^-iBECs. N ​= ​2 biological replicates and a minimum of n ​= ​3 for independent replicates per line. Statistical analysis was completed by using one-way-ANOVA, error bars ​= ​SD.∗P ​< ​0.05.

**Fig. S8:**
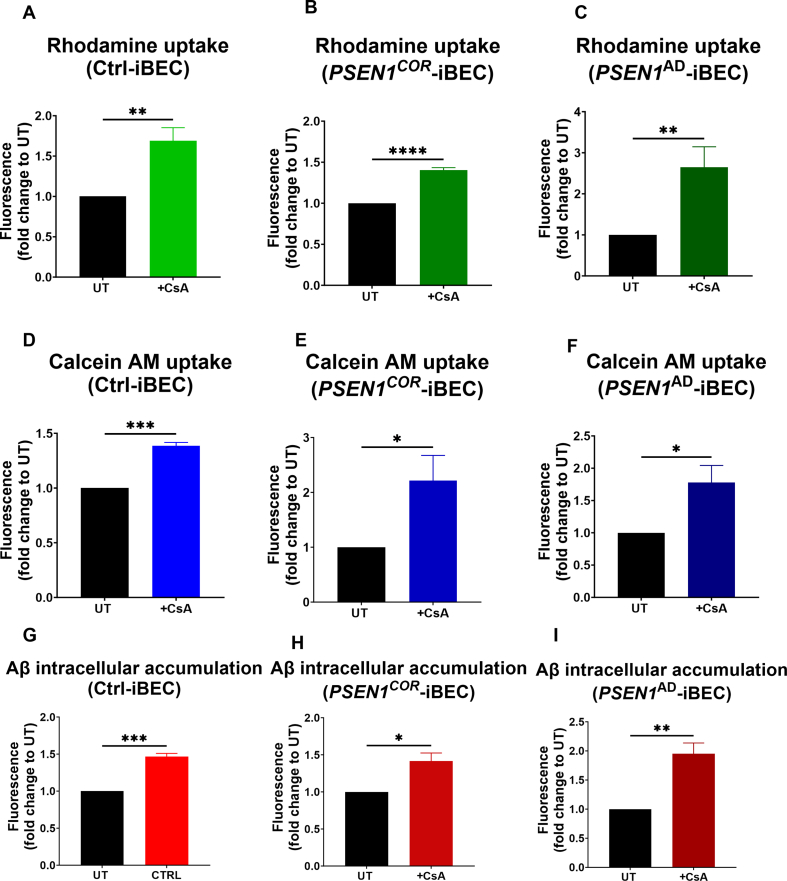
Characterization of functional activity of P-gp, MRP1 and P-gp-mediated amyloid-β uptake in untreated Ctrl-, *PSEN1*^cor^- and *PSEN1*^AD^-iBECs. Intracellular fluorescence of (A–C) rhodamine 123 in Ctrl-, *PSEN1*^*cor*^ and *PSEN1*^AD^-iBECs without (UT) or with (+CsA) P-gp inhibition with CsA. Intracellular fluorescence of (D – F) calcein-AM in Ctrl-, *PSEN1*^cor^ and *PSEN1*^AD^-iBECs without (UT) or with (+MK-571) MRP1 inhibition with MK-571. Intracellular fluorescence of (G–I) FITC-amyloid β in Ctrl-, *PSEN1*^cor^ and *PSEN1*^AD^-iBECs without (UT) or with (+CsA) P-gp inhibition with CsA. In all lines an increase in uptake of over 30–40 ​% was seen when treated with inhibitor compared to UT, suggesting functional presence of the studied transporters. N ​= ​2 biological replicates and a minimum of n ​= ​3 for independent replicates per line. Statistical analysis was completed by using Student's test, error bars ​= ​SEM. ∗P ​< ​0.5 and ∗∗∗P ​< ​0.001.

The analysis of the remaining SLC transporters investigated in this study (*SLC22A3* (OCT3), *SLC22A8* (OAT3), *SLC7A5* (LAT1), demonstrated minimal changes following FUS treatments (Fig. S7). *SLC22A8* was downregulated immediately following FUS^+MB^ treatment in *PSEN1*^COR^ iBECs compared to UT (Fig. S7B) and *SLC7A5* was downregulated in *PSEN1*^COR^ iBECs 24 ​h after FUS^+MB^ when compared to UT (Fig. S7C). These results suggest mild modulatory effects of FUS on SLC transporter expression, with the potential to increase LRP1 expression, observed to be downregulated in AD. The findings are summarized in Table S5.

### Effects of FUS treatment on P-gp activity in control and *PSEN1*^AD^-iBECs

**Fig. 4:**
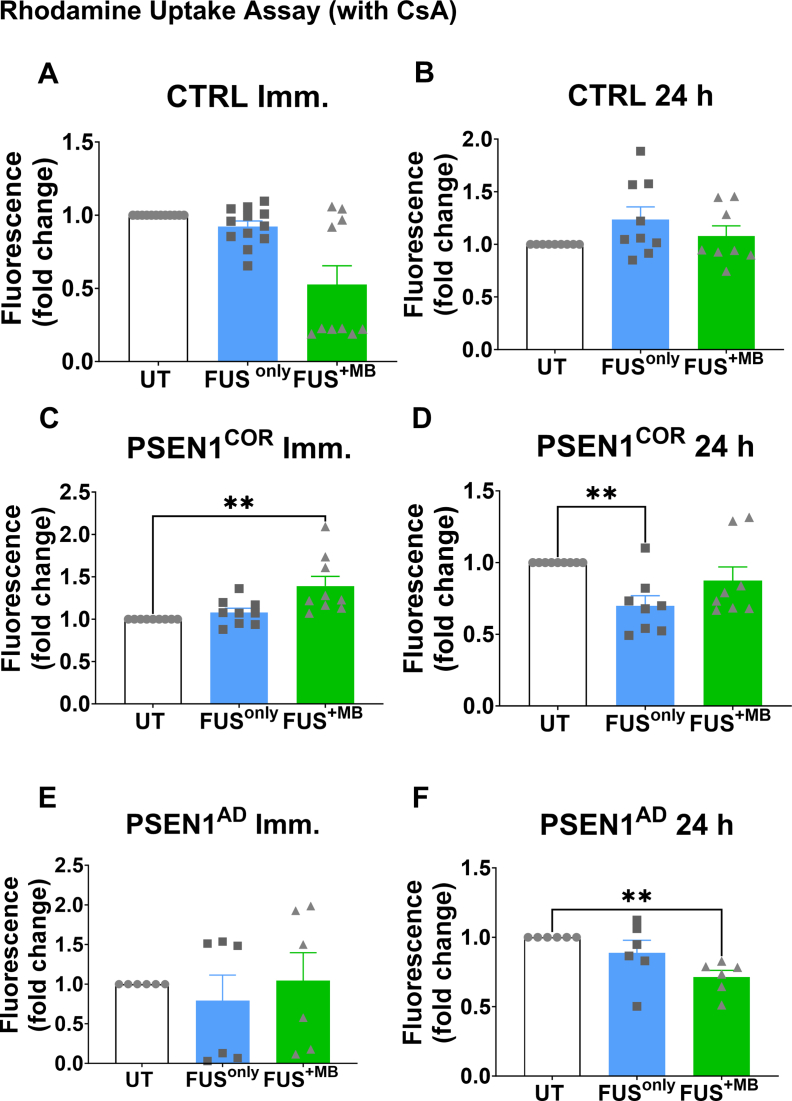
**P-gp activity measured by rhodamine 123 accumulation in Ctrl-, *PSEN1*^*COR*^*-* and *PSEN1*^AD^-iBECs following treatment with FUS^only^ or FUS^+MB^**. (A to F) P-gp activity measured by rhodamine 123 uptake immediately and at 24 ​h in UT, FUS^only^ and FUS^+MB^ conditions following CsA treatment in (A-B) Ctrl-iBECs, (C-D) *PSEN1*^*COR*^-iBECs and (E-F) *PSEN1*^AD^-iBECs. Results are indicated as fold change to UT, N ​= ​2 biological replicates and a minimum of n ​= ​3 for independent replicates per line. Statistical analysis was completed by using one-way-ANOVA, error bars ​= ​SEM.∗∗P ​< ​0.01.

**Fig. S9:**
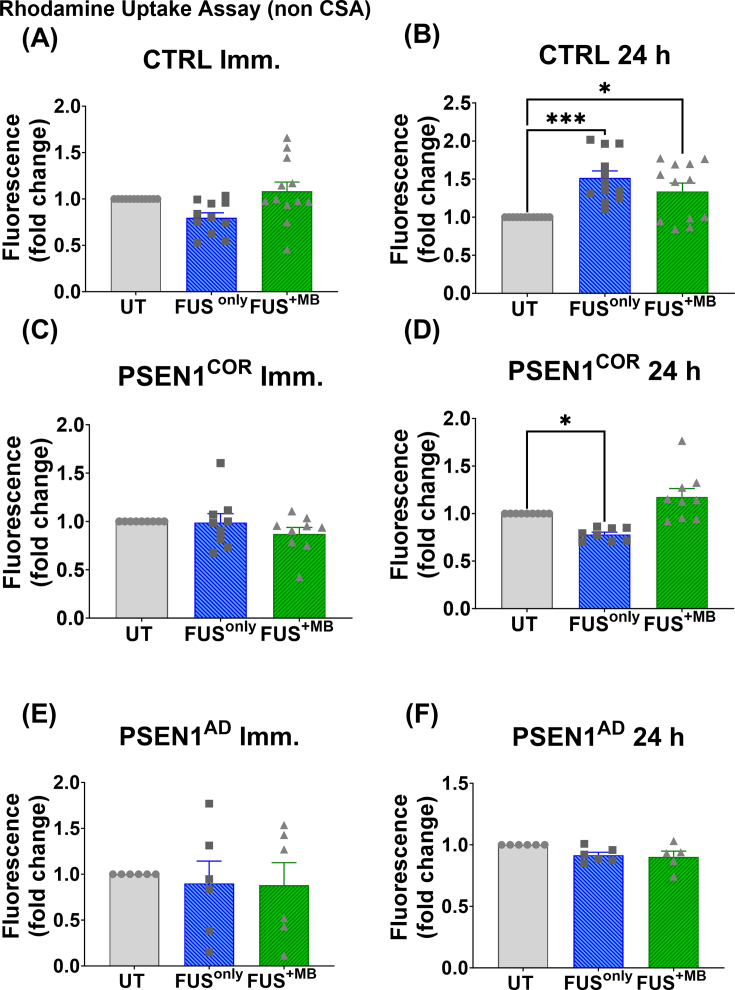
P-gp activity measured by rhodamine 123 accumulation in Ctrl, *PSEN1*^COR^ and *PSEN1*^AD^-iBECs in the absence of CsA (non CsA) following treatment with FUS^only^ or FUS^+MB^. (A to F) P-gp activity measured by rhodamine 123 uptake immediately and at 24 ​h in UT, FUS^only^ and FUS^+MB^ conditions in the absence of CsA treatment in (A-B) Ctrl-iBECs, (C-D) *PSEN1*^*COR*^-iBECs and (E-F) *PSEN1*^AD^-iBECs. Results are indicated as fold change to UT, N ​= ​2 biological replicates and a minimum of n ​= ​3 for independent replicates per line. Statistical analysis was completed by using one-way-ANOVA, error bars ​= ​SEM.∗P ​< ​0.05 and ∗∗∗P ​< ​0.001.

As gene expression does not necessarily equate directly to functional activity of drug transporters, we next examined the effects of FUS^only^ and FUS^+MB^ on P-gp functional activity in iBECs. To achieve this, we measured the intracellular accumulation of the P-gp substrate rhodamine 123 following FUS^only^ and FUS^+MB^ treatment after performing P-gp inhibition with CsA (Fig. 4), and without P-gp inhibition (non-CSA, Fig. S9) a common assay used to measure P-gp activity [39,55,80,81].

When we examined the effects of CsA treatment followed by FUS treatments (Fig. 4), our results revealed that in Ctrl-iBECs no differences in rhodamine 123 accumulation were identified following FUS treatments compared to UT (Fig. 4A & B). In contrast, for *PSEN1*^*COR*-^iBECs the accumulation of rhodamine 123 was significantly increased immediately after FUS^+MB^ when compared to UT (Fig. 4B), and after 24 ​h rhodamine 123 accumulation was significantly decreased following FUS^only^ when compared to UT (Fig. 4B), suggesting potential modulatory effects on P-gp activity. Similar to Ctrl-iBECs, rhodamine 123 accumulation was not altered in *PSEN1*^AD^-iBECs immediately after FUS treatments when compared to UT (Fig. 4B), but at 24 ​h, rhodamine 123 accumulation was significantly decreased in FUS^+MB^ treated condition compared to UT, suggesting increased P-gp activity (Fig. 4B).

Our data from non-CsA-treated samples showed that 24 ​h following FUS treatments Ctrl-iBECs exhibited lower activity of P-gp, indicated by significantly increased rhodamine uptake (Fig. S9B). In contrast, and consistent with CsA treated cells, *PSEN1*^COR^-iBECs demonstrated increased P-gp activity 24 ​h following FUS^only^ treatment, indicated by significantly decreased rhodamine 123 uptake (Fig S9D). In contrast, no significant changes in rhodamine 123 uptake were observed in non-CsA treated *PSEN1*^AD^-iBECs following either of the FUS treatments at either timepoint (Fig. S9C).

Overall, our data suggests that FUS treatments modulates P-gp functional activity in a patient-and disease-specific manner.

### Effects of FUS treatment on MRP1 activity in control and *PSEN1*^AD^-iBECs

To assess MRP1 activity following FUS^only^ and FUS^+MB^ treatments we used a calcein-AM uptake assay. Calcein-AM is an MRP1 substrate dye that has been extensively used to measure MRP1 activity, together with the MRP1 inhibitor MK-571 [57,82]. Similar to the rhodamine 123 uptake, we measured calcein-AM uptake in the absence and presence of the MRP1 inhibitor MK-571 for all groups (UT, FUS ^only^ and FUS^+MB^) immediately and 24 ​h post treatment, respectively.

**Fig. 5:**
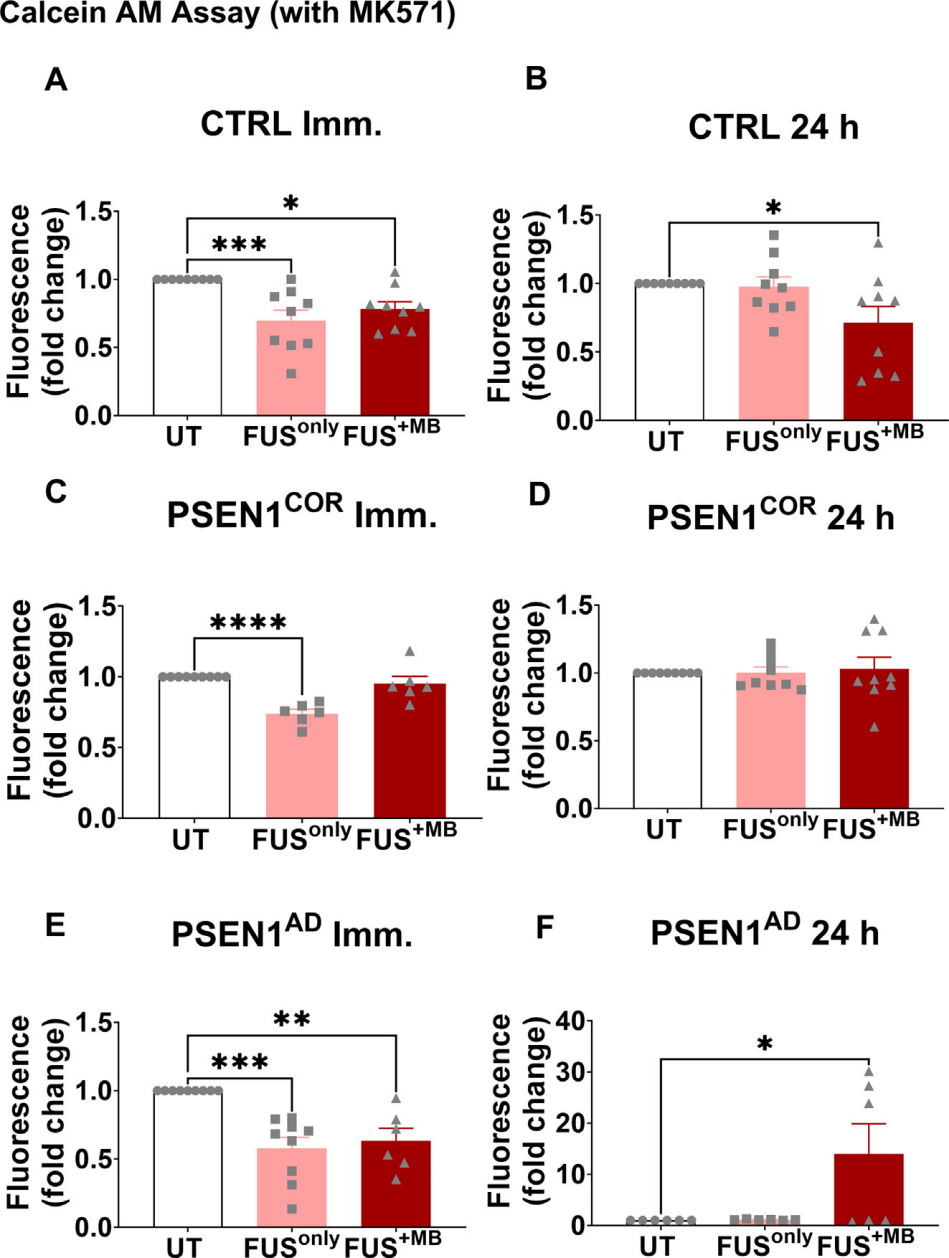
**MRP1 activity measured by Calcein-AM accumulation in Ctrl-, *PSEN1*^*COR*^*-* and *PSEN1*^AD^-iBECs following treatment with FUS^only^ or FUS^+MB^**. (A to F) MRP1 activity measured by calcein-AM uptake immediately and at 24 ​h in UT, FUS^only^ and FUS^+MB^ conditions following MK-571 treatment in (A–B) Ctrl-iBECs, (C–D) *PSEN1*^*COR*^-iBECs and (E–F) AD-iBECs. Results are indicated as fold change to UT, N ​= ​2 biological replicates and a minimum of n ​= ​3 for independent replicates per line. Statistical analysis was completed by using one-way-ANOVA, error bars ​= ​SEM. ∗P ​< ​0.05, ∗∗P ​< ​0.01, ∗∗∗P ​< ​0.001 and ∗∗∗∗P ​< ​0.0001.

When the samples subjected to MK-571 inhibition were analyzed followed by FUS treatment, our data revealed that Calcein-AM uptake was significantly decreased in Ctrl-iBECs immediately after FUS^only^ and FUS^+MB^ treatments compared to UT (Fig. 5A), suggesting increased MRP1 activity. Reduced Calcein-AM uptake following FUS^+MB^ was maintained in Ctrl-iBECs at the 24 ​h timepoint (Fig. 5B). Similar to Ctrl-iBECs, decreased Calcein-AM uptake was also observed in *PSEN1*^COR^-iBECs following FUS^only^ at the immediate timepoint, however, no effects were seen for FUS^+MB^ in either timepoint (Fig. 5C–D), Interestingly, similar effects were seen for *PSEN1*^AD^-iBECs as for the Ctrl-iBECs, with the accumulation of Calcein-AM significantly reduced compared to UT immediately after FUS^only^ and FUS^+MB^ treatments, suggesting a higher activity of MRP1 (Fig. 5E–F). However, in contrast to Ctrl-iBECs, Calcein-AM uptake was significantly increased 24 ​h following FUS^+MB^, indicating decreased MRP1 activity at the later timepoint (Fig. 5F).

**Fig. S10:**
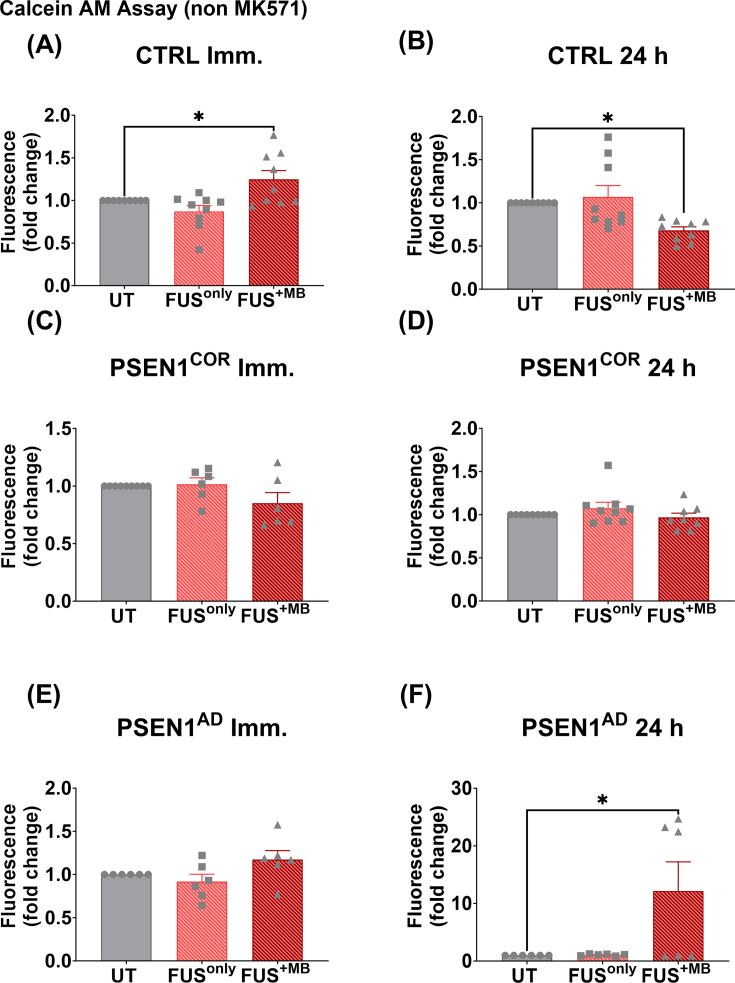
MRP1 activity measured by Calcein AM uptake in Ctrl, *PSEN1*^COR^ and *PSEN1*^AD^-iBECs in the absence of MK571 (non MK571) following treatment with FUS^only^ or FUS^+MB^. (A to F) MRP1 activity measured by Calcein AM uptake at immediately and 24 ​h in UT, FUS^only^ and FUS^+MB^ conditions in the absence of MK571 treatment in (A-B) Ctrl-iBECs, (C-D) *PSEN1*^*COR*^-iBECs and (E-F) *PSEN1*^AD^-iBECs. Results are indicated as fold change to UT, N ​= ​2 biological replicates and a minimum of n ​= ​3 for independent replicates per line. Statistical analysis was completed by using one-way-ANOVA, error bars ​= ​SEM.∗P ​< ​0.05.

When the analysis of MRP1 activity was conducted in samples without the MK-571 inhibitor, the results revealed notable differences. In Ctrl-iBECs, there was a higher accumulation of Calcein AM immediately after FUS^+MB^ treatments, suggesting lower MRP1 activity (Fig. S10A). However, after 24 ​h, the effects appeared to reverse, suggesting a lower accumulation of Calcein AM followed by FUS^+MB^ treatment which consequently indicates higher MRP1 activity (Fig. S10B). For *PSEN1*^COR^-iBECs, there were no significant changes observed at either timepoint irrelevant of the FUS treatment applied (Fig. S10C-D). In the case of *PSEN1*^AD^-iBECs, changes seemed to occur specifically at 24 ​h after FUS^+MB^ treatment, where a higher accumulation of Calcein AM was found, consistent with MK-571 treated cells. This suggests that FUS^+MB^ treatment may have a downregulatory effect on MRP1 activity in *PSEN1*^*AD*^-iBECs (Fig. S10E).

Altogether, these results indicate that FUS can modulate the functional activity of MRP1, and the results appear to be more robust in the *PSEN1* mutant phenotype, suggesting that the diseases-onset and different phenotypes should be considered when FUS is used for therapy.

### Effects of FUS treatment on P-gp-mediated Aβ uptake in control and *PSEN1*^AD^-iBECs

We then investigated whether FUS^only^ or FUS^+MB^ treatment could modulate P-gp-mediated Aβ uptake. P-gp has been reported to play a role in Aβ clearance [83] and P-gp expression is reported to be altered in AD patients, [34,84]. To confirm that P-gp mediates uptake of Aβ in iBECs, we performed P-gp inhibition via CsA, which resulted in increased Aβ uptake (Fig. S6G-I).

**Fig. 6:**
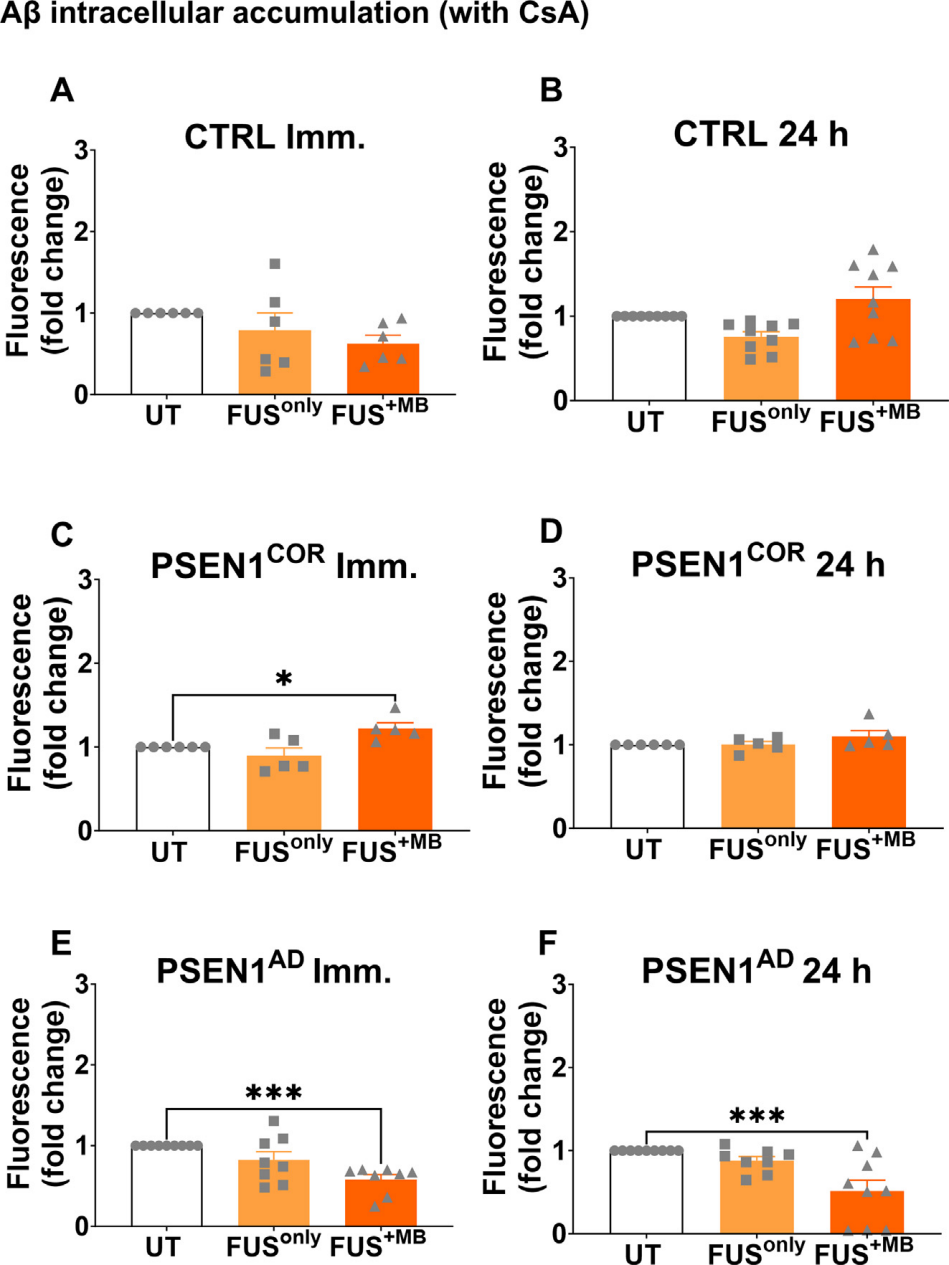
**P-gp-mediated Aβ uptake in Ctrl-iBEC, *PSEN1*^*COR*^-iBEC and *PSEN1*^AD^-iBEC in UT and after treatment with FUS**. Aβ uptake measured by fluorescence immediately and 24 ​h following in FUS^only^ or FUS^+MB^ treatments compared to UT in CsA inhibited (A & B) Ctrl-iBECs, (C & D) *PSEN1*^*COR*^-iBECs and (E & F) *PSEN1*^AD^-iBECs. Results are indicated as fold change to UT, N ​= ​2 biological replicates and a minimum of n ​= ​3 for independent replicates per line. Statistical analysis was completed by using one-way-ANOVA, error bars ​= ​SEM. ∗P ​< ​0.05 and ∗∗∗P ​< ​0.001.

When analyzed in the presence of CsA we observed no changes in intracellular Aβ accumulation in Ctrl-iBECs following FUS treatments at immediate or 24 ​h timepoints (Fig. 6A and B). In contrast, in *PSEN1*^*COR*^-iBECs, Aβ accumulation was significantly increased immediately following FUS^+MB^ treatment (Fig. 6C). However, at the 24 ​h timepoint no significant effects of FUS treatments on Aβ accumulation were identified in *PSEN1*^*COR*^-iBECs. Interestingly, in *PSEN1*^AD^-iBECs a consistent effect following FUS^+MB^ treatment was seen with a significant reduction in Aβ accumulation identified immediately and 24 ​h following FUS^+MB^ treatment compared to UT, suggesting a decrease in P-gp-mediated Aβ uptake (Fig. 6E and F).

**Fig. S11:**
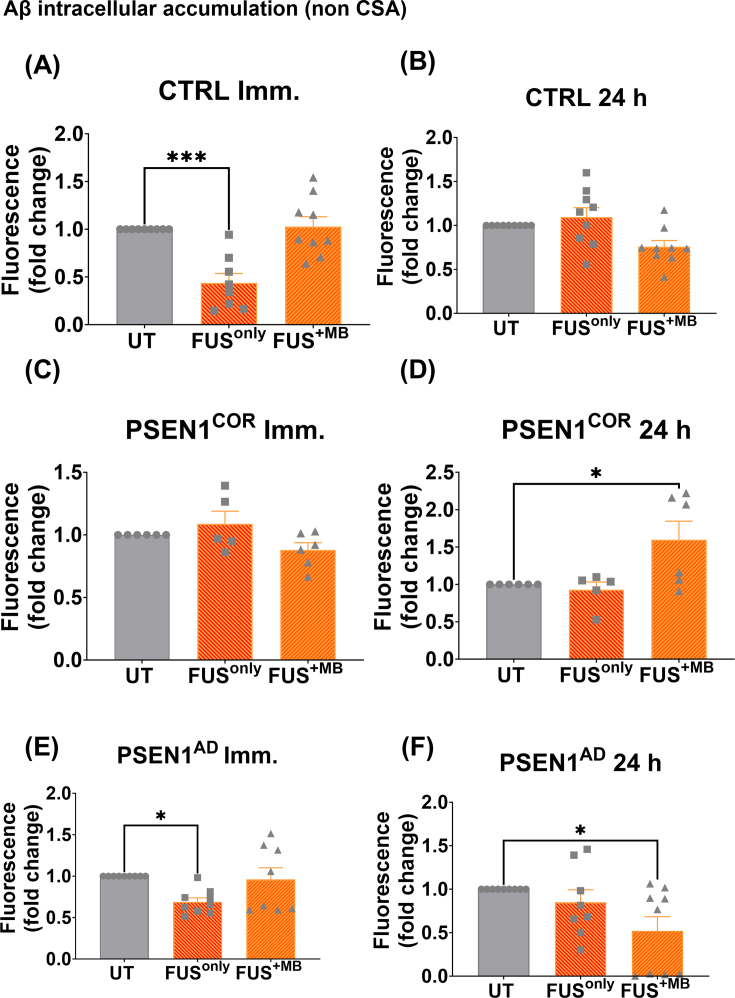
P-gp-mediated Aβ uptake in Ctrl-iBEC, *PSEN1*^*COR*^-iBEC and *PSEN1*^AD^-iBEC in in the absence of CsA following treatment with FUS^only^ or FUS^+MB^. (A-F) Aβ uptake measured by fluorescence immediately and 24 ​h following in FUS^only^ or FUS^+MB^ treatments compared to UT in CsA inhibited (A & B) Ctrl-iBECs, (C & D) *PSEN1*^*COR*^-iBECs and (E & F) *PSEN1*^AD^-iBECs. Results are indicated as fold change to UT, N ​= ​2 biological replicates and a minimum of n ​= ​3 for independent replicates per line. Statistical analysis was completed by using one-way-ANOVA, error bars ​= ​SEM. ∗P ​< ​0.05 and ∗∗∗P ​< ​0.001.

Following this, a comparison of Aβ uptake in the absence of CsA followed by FUS treatments were conducted. Our results from the non-CsA group showed that in Ctrl-iBECs there was a lower intracellular accumulation of Aβ observed immediately following FUS^only^ (Fig. S11A&E). In contrast, at the 24 ​h time point, higher Aβ accumulation was observed in *PSEN1*^COR^-iBECs after FUS^only^ treatment (Fig. S11D). Interestingly, in *PSEN1*^AD^-iBECs, we observed a reduced intracellular accumulation of Aβ in samples analyzed immediately following FUS^only^ and 24 ​h after FUS^+MB^ treatment (Fig. S11E&F).

The results observed in Aβ intracellular accumulation following modulation by FUS treatments suggest the ability to modulate both P-gp-dependent and P-gp independent cell Aβ uptake/clearance with FUS.

## Discussion

Transporters at the BBB play a vital role in regulating the entry of molecules from the blood to the brain, but some, such as efflux pumps, challenge the delivery of drugs into the brain [35]. In addition, in brain disorders such as AD, transporter activity is altered, potentially contributing to disease progression [85,86]; however the full extent of AD-related changes in BBB transporters in human are not known. As such, the contribution of BBB transporters in AD pathogenesis as well as their effects on drug delivery can be a major impediment to drug therapy. As previously reported by our group [39,42] and others, BBB opening by FUS^+MB^ is a novel technology that is becoming a promising tool to increase drug delivery into the brain [87,88]. In addition, FUS^only^ can have modulatory effects on the brain that are not well understood [39,86]. However, the molecular effects of FUS^only^ and FUS^+MB^ on human BBB transporters are still unknown with these treatments potentially providing a means to modulate transporter activity.

In this study, we employed hiPSC-derived iBECs as a model for the investigation of BBB function. To confirm the suitability of our model for BBB transporter research, we compared iBECs to a human immortalized microvascular endothelial cell line, hCMEC/D3. Our findings highlight that both iBECs and hCMEC/D3, exhibit the expression of crucial BBB markers, including TJs and transporters, being in-line with previous studies [49,[89], [90], [91]]. Although our results demonstrate that iBECs contain a significant level of epithelial marker expression, as also previously demonstrated [60], minimal differences were found in the levels of BBB transporter expression between iBECs and hCMEC/D3, suggesting the suitability of iBECs for BBB transporter research. Furthermore, iBECs allow for the study of disease- and patient-specific effects, not possible using hCMEC/D3 or other human BEC lines.

Following iBEC characterisation in comparison to hCMEC/D3 we first performed a detailed investigation of the expression of 12 BBB transporters known to be related to AD or BBB dysfunction using our previously reported familial iBEC *in vitro* model [39]. We then used this model to investigate the effects of FUS^only^ and FUS^+MB^ on BBB transporter expression and activity to identify potential novel modulatory effects of therapeutic FUS.

Of the studied transporters, our results demonstrated that the relative gene expression of *ABCC1* (MRP1)*, ABCG2* (BCRP) *SLCO1A2* (OATP12) and *LRP1* was significantly downregulated in *PSEN1*^COR^ and/or *PSEN1*^AD^-iBECs compared to Ctrl-iBECs suggesting potentially reduced activity in FAD patients irrespective of the *PSEN1* mutation. Interestingly, some of these findings were not in-line with our previous study [39], which in contrast showed increased expression for MRP1 and BCRP in AD-iBECs compared to Ctrl-iBECs. In our previous study a different composition of cell lines was used, for example one Ctrl-iBEC and two *PSEN1*^COR^-iBEC lines were pooled into a combined Ctrl-iBEC group and the *PSEN1*^AD^-iBECs contained an additional iBEC line that was not used in the current study (because this line did not have an isogenic *PSEN1* corrected control). As such, it is important to acknowledge that one limitation in using patient-derived hiPSCs is line to line variation, which might affect experimental outcomes. Of note, although not significant, our findings in the present study show a higher trend in expression of BCRP in *PSEN1*^AD^-iBECs compared to *PSEN1*^COR^-iBECs, suggesting an association with the *PSEN1* mutation with BCRP expression.

Interestingly, our results suggest a link between *PSEN1* mutation and transporters involved in Aβ transport. A previous study has shown that low levels of *ABCC1* (MRP1), might correlate with increased levels of Aβ40 and Aβ42 in the CNS [64], potentially contributing to the formation of Aβ plaques. In addition, LRP1 is also involved in Aβ transport, thus the changes in the expression of this transporter can directly impact the clearance of Aβ from the brain, contributing to the neuropathology of AD [30,75,76,92,93]. Thus, the simultaneous downregulation of the two genes *ABCC1* (MRP1) and *LRP1* as seen in our model, could potentially result in increased Aβ plaque formation, enhancing FAD progression. As such, our results indicate that patient cell-based models may offer a promising approach to study AD-related BBB dysfunction.

Another important finding from the screening was the changes observed on the *SLCO1A2* (OATP12) which is an important Solute Carrier Organic Anion Transporter (SLCO) in the human brain, highly expressed in the brain and in specific regions, such as BBB, where it plays a major role in uptake of drugs into the brain [63]. The downregulation of OATP12 observed in our *in vitro* model for FAD could potentially impact the disease by impairing the transport of certain substrates across the BBB potentially altering the levels of neuroactive compounds or neurotoxic substances in the brain, which may contribute to AD pathology or progression. Considering the important functions of OATP12 in maintaining brain homeostasis [27,69], investigating its roles at a more in-depth level in relation to AD could potentially identify new therapeutic targets.

Because BBB-specific transporters provide potential therapeutic targets in AD, there is an interest in identifying whether we can modulate their activity. We have previously reported the ability to model FUS^+MB^-mediated BBB opening using the same patient-derived iBEC model as in the current study [39,42]. We have also previously demonstrated that the mRNA levels of TJPs are altered following FUS^+MB^ [39], however, the effect of FUS^only^ and FUS^+MB^ on BBB transporter function remains sparsely uninvestigated. Thus, we screened the 12 BBB studied transporters in Ctrl-, *PSEN1*^*COR*^- and *PSEN1*^AD^-iBECs following FUS^only^ and FUS^+MB^ treatments.

In most transporters, we did not observe significant effects following FUS treatments, however, interesting effect were observed for *ABCB1* (P-gp), *ABCC1* (MRP1) and *LRP1*, with *ABCC1*, and *LRP1* demonstrating dysfunctional expression in *PSEN1*^*AD*^*-*iBECs following our initial screening. Although we did not identify significant differences in *ABCB1* (P-gp) expression following our initial screening, the upregulation of P-gp following FUS treatments is interesting, as P-gp plays a key roles in drug efflux and Aβ clearance [[94], [95], [96]], thus modulation of P-gp via FUS could have therapeutic effects. For *ABCC1* (MRP1) FUS^only^ and FUS^+MB^ appeared to have an AD (non *PSEN1*-related) response to FUS^only^ and FUS^+MB^ as both *PSEN1*^*COR*^ and *PSEN1*^AD^-iBECs demonstrated a strong downregulation in *ABCC1* (MRP1) expression immediately following treatments, not observed in Ctrl-iBECs. These results indicate a potential patient-specific response to FUS^only^ and FUS^+MB^ and that some transporters might be differentially altered following FUS treatments depending on the disease status. The strong downregulation in *ABCC1* (MRP1) expression following FUS treatments in *PSEN1*^*COR*^ and *PSEN1*^AD^-iBECs also suggests that there is a potential to downregulate *ABCC1* (MRP1) gene expression during a short window following treatment. The downregulated gene expression of MRP1 following FUS^+MB^ in *PSEN1*^AD^-iBECs also correlated with the functional assay, which showed decreased MRP1 activity (increased calcein-AM uptake) 24 ​h following FUS^+MB^ treatment. This likely suggests that effects of FUS on MRP1 gene expression do not immediately reflect to MRP1 functionality. Overall, Our results also demonstrate that the effects of FUS^+MB^ on MRP1 gene expression are transient, likely suggesting only transient effects on functionality, which is in-line with the known effects of FUS by us and others [39,42,97]. These findings hold importance as they suggest that in cases where MRP1 hinders the entry of specific drugs into the brain, modifying its expression with FUS may offer a small window of opportunity to deliver these drugs [98,99].

Intriguingly, our findings strongly suggest that FUS treatments were able to increase *LRP1* expression in *PSEN1*^COR^ and *PSEN1*^AD^-iBECs, which both demonstrated reduced *LRP1* expression compared to Ctrl-iBECs. These findings are significant because, LRP1 plays a role in regulating the levels of Aβ and controlling the levels and spread of tau protein in the brain [75]. Previous studies have reported decreased in LRP1 levels in normal aging processes and in AD pathogenesis [76], further confirmed by our results. Our results also suggest that FUS treatments could be used to re-establish LRP1 levels in AD patients. However, it is important to note that in this study, the activity of LRP1 was not explored. Further investigation is needed to assess the FUS modulatory effects on the activity of BBB transporters mediated via the RMT superfamily, which may offer an interesting therapeutic avenue [4,7,100].

Finally, we used our *in-vitro* iBEC model to investigate changes P-gp-mediated Aβ uptake following FUS^only^ and FUS^+MB^ to establish a potential link between FUS-mediated transporter modulation and Aβ clearance. Interestingly, our results suggest that in *PSEN1*^AD-^iBECs FUS^+MB^ increased P-gp activity (indicated by decreased rhodamine 123 uptake), which was associated with decreased cellular uptake of Aβ, suggesting increased Aβ clearance from cells. As such, our data suggests that P-gp is likely involved in Aβ clearance, which could potentially be increased with FUS^+MB^, with some disease types potentially more susceptible to these effects as this effect was not seen in Ctrl- and *PSEN1*^*cor*^-iBECs. The effect of decreased Aβ uptake in *PSEN1*^AD-^iBECs was also seen in the absence of P-gp inhibition, suggesting also non-P-gp-associated pathways might play a role in this process. To confirm that the decreased cellular Aβ uptake also correlates with increased Aβ clearance from the brain, cell culture media analysis of soluble Aβ would be needed. However, in our previous study using the same *PSEN1*^AD^-iBECs in a Transwell culture setting, we demonstrated persistent Aβ clearance across the *PSEN1*^AD^-iBEC barrier following FUS^+MB^, supporting the findings of the current study [39].

Our findings emphasize the potential to enhance Aβ clearance as well as Aβ transporting transporters in *PSEN1*^AD^-iBECs following FUS^+MB^, which provides a potential new avenue of therapeutic intervention. Nevertheless, it is important to acknowledge that previous studies have shown increased permeability in animal and human models for both natural aging process and AD [101,102], which might indicate that the FUS^+MB^ treatments could potentially exacerbate this permeability. Importantly, a recent *in vivo* mouse model study indicated that the increases on the BBB permeability of aged and AD mouse does affect the duration of BBB opening and closure followed by FUS^+MB^ [103]. Increased and prolonged permeability could elicit other downstream cascades, such as increased inflammation [104]. Thus, the potential adverse effects of FUS treatments should be taken into account when adjusting FUS parameters for the treatment of individual AD patients.

In conclusion, our findings demonstrate that *PSEN1* mutant *PSEN1*^AD^-iBECs possess phenotypical differences compared to control iBECs in BBB transporter expression and function, which further supports the contribution of FAD mutations on BBB dysfunction. Additionally, we show for the first time in a human AD BBB cell model that FUS^only^ and FUS^+MB^ can modulate the BBB transporter expression and functional activity in iBECs, having potential implications on drug penetration and amyloid clearance offering important considerations for therapeutic design of FUS in modulating BBB transporters for drug delivery interventions to treat AD. Our results further highlight the differential responses of patient cells to FUS treatment, with patient-derived models likely providing an important tool for modelling therapeutic responses of FUS in a more individualized manner depending on the phenotype to make the translation of FUS more effective to humans.

## Author contributions

J.C.S.C and L.E.O designed the project. J.C.S.C performed most experiments, analyzed the data, and wrote the manuscript. J.M.W provided technical assistance with the ultrasound experiments. C.C.L and L.M.R assisted in sample processing. S.L provided technical assistance in functional assays and immunofluorescence experiments. J.K provided the iPSC lines used in this study. L.E.O and A.R.W interpreted the data, reviewed and edited the manuscript.

## Ethics approval

This study has been approved by the QIMR Berghofer Medical Research Institute Human Ethics Committee (approval P21970).

## Availability of data and materials

Data used to obtain the results presented in the manuscript can be obtained from the corresponding author upon reasonable request.

## Funding

This research was supported by QUT Postgraduate Research Award (QUTPRA) and QUT Higher Degree Research Tuition Fee Scholarship 2020 (to J.C.S.C). This research was supported by QIMR Berghofer SEED grant (to L.E.O), NHMRC Project grants APP1125796 (to A.R.W.). A.R.W. is a recipient of an NHMRC Senior Research Fellowship (APP1118452). This project was supported through the Academy of Finland under the aegis of JPND—www.jpnd.eu––and European Union's Horizon 2020 research and innovation program under grant agreement no. 643417 (to J.K).

## Declaration of competing interest

The authors declare no competing interests.
