# Supplementary material for: Alzheimer's disease brain endothelial-like cells reveal differential drug transporter expression and modulation by potentially therapeutic focused ultrasound
Source: Neurotherapeutics. 2023 Dec 19;21(1):e00299. doi: 10.1016/j.neurot.2023.10.009 (PMC10903103; doi:10.1016/j.neurot.2023.10.009)
Supplement: Multimedia component 1 [file mmc1.docx]

**Table S1- Further information of hiPSC lines used in this study.**

| **Cell ID** | **Gender** | **Age at Biopsy** | **Line** | **Ctrl, FAD or AD** | **Karyotype** | **Mycoplasma** |
| --- | --- | --- | --- | --- | --- | --- |
| HDFa | F | unknown | Control | Control | normal | negative |
| Ctrl 8.2 |  | unknown | Control | Control | normal | negative |
| AD4 1.6 | M | 48 | AD1-*PSEN1* | Familial | normal | negative |
| AD4 1.6.1.2.9 | M | 48 | AD1-*PSEN1^COR^* | Familial | normal | negative |
| AD5.1.5 | F | 47 | AD2-PSEN1 | Familial | normal | negative |
| AD5.1.5.6.1 | F | 47 | AD2-*PSEN1^COR^* | Familial | normal | negative |

**Table S2 – Immunofluorescence antibodies used for hiPSC and iBEC characterization.**

| **Antibody** | **Host Species** | **Antibody Target** | **Fixation** | **Dilution** | **Supplier and Catalogue Number** |
| --- | --- | --- | --- | --- | --- |
| **Primary Antibodies** | | | | | |
| **SOX2** | **Rat** | **Pluripotent cell marker**  **(Stem cells marker)** | **4%PFA** | **1:100** | **Thermo Fisher Scientific**  **cat# 14-9811-82** |
| **Nanog** | **Rabbit** | **Pluripotent cell marker**  **(Stem cells marker)** | **4%PFA** | **1:100** | **Abcam**  **cat#ab21624** |
| **Occludin** | **Rabbit** | **Tight Junction protein (BBB marker)** | **Methanol** | **1:100** | **Thermo Fisher Scientific**  **cat# 71-1500** |
| **Claudin5** | **Mouse** | **Tight Junction protein (BBB marker)** | **Methanol** | **1:100** | **Thermo Fisher Scientific**  **cat# 35-2500** |
| **ZO1** | **Mouse** | **Tight Junction protein (BBB marker)** | **Methanol** | **1:100** | **Thermo Fisher Scientific**  **Cat#33-9100** |
| **VE-Cadherin** | **Rabbit** | **Junction Adherent Protein**  **(BBB marker)** | **4% PFA** | **1:50** | **Sigma**  **cat#MABT886** |
| **GLUT1** | **Mouse** | **Glucose transporter**  **(Transporter marker)** | **Methanol** | **1:200** | **Thermo Fisher Scientific**  **cat# MAS-11315** |
| **Secondary Antibodies** | | | | | |
| **Goat anti-rabbit IgG (H+L) Alexa Fluor 488** | | |  | **1:1000** | **Thermo Fisher Scientific**  **cat#A-27034** |
| **Goat anti-mouse IgG (H+L) Alexa Fluor 594** | | |  | **1:1000** | **Thermo Fisher Scientific**  **cat#A-11032** |
| **Goat- anti Rat IgG (H+L) Alexa Fluor 647** | | |  | **1/1000** | **Thermo Fisher Scientific**  **cat#A-21247** |

**Table S3: qPCR primer sequences for all transporters that were used in this study.**

| **Target** | **Forward Sequence** | **Reverse Sequence** |
| --- | --- | --- |
| *Occludin* | GAAGCAAGTGAAGGGATCTGC | ACAACTTGGCATCAGCCTTCT |
| *Claudin 5* | GATTGAGAGGTCTGGGAAGCC | ATCCCATGGCAAACAGAGAGG |
| *ZO1* | ACAGCTACAGGAAAATGACCGA | ACTGGTTCAGGATCAGGACG |
| *VE-Cadherin* | AGGCAAGATCAAGTCAAGCGT | GAGTCTCCAGGTTTTCGCCA |
| *PECAM* | GACGTGCAGTACACGGAAGTT | GGGAGCCTTCCGTTCTAGAGTAT |
| *SOX18* | TCAGCAAGATGCTGGGCAAAG | GCGGCCGGTACTTGTAGTTG |
| *EPCAM* | CGCAGCTCAGGAAGAATGTGT | ACCAACTGAAGTACACTGGCA |
| *SLC2A1* = GLUT1 | TGGCATCAACGCTGTCTTCT | ACAACTTGGCATCAGCCTTCT |
| *SLCO1A2 =* OATP12 | CACCCTGAAGAGCAACATGG | TTGCCAACAGAAACATCTTCAAC |
| *SLC22A8 =* OAT3 | TCCATTGTGACAGAGTGGGACT | GACAGGTCTCCAAGCACGAG |
| *SLC7A5 =*LAT1 | TTATCGGCTCGGGCATCTTC | CCTCCAGCATGTAGGCGTAG |
| *SLC22A3 =* OCT3 | CACCATCGTCAGCGAGTTTGA | GCCAACACCAAGGCAGGATA |
| *ABCB1 =* P-gp | CAGATAAAAGAGAGGTGCAACGG | GCCCGGATTGACTGAATGTT |
| *ABCG2 =* BCRP | TGTTTTGTGTTTATGATGGTCTGT | GCTGCAAAGCCGTAAATCCAT |
| *ABCG4 =* ATPG4 | AGACCCTTCTCAAGTGCCTCT | CCTTCACAGACTCCCTGTATC |
| *ABCC1 =* MRP1 | TGCTGGGCAGACCTCTTCTA | AAAAAGGTAGCAAGCAGCATGG |
| *ABCC2 =* MRP2 | CTGTCACCAGGTCCCCAATC | CGAATTGCAAGCCACCTGTT |
| *ABCA1 =* Cholesterol | ACAGTTAATGACCAGCCACGG | AGCAGCAGCTGACATGTTTGT |
| *LRP1 =* LDL receptor | AGTCTGCTTCGTGCCTATC | CAGTCATTGTCATTGTCGCATCT |
| *18S* | TTCGAGGCCCTGTAATTGGA | GCAGCAACTTTAATATACGCTATTGG |

**Table S4: Summary of the comparison in expression of BBB drug transporters screened in Fig 1**

| **CTRL vs *PSEN1^COR^*** | **CTRL vs *PSEN1*^AD^** | ***PSEN1^COR^* vs *PSEN1^A^*^D^** |
| --- | --- | --- |
| *ABCA1* | *ABCA1* | *ABCA1* |
| *ABCB1* (PGP) | *ABCB1* (PGP) | *ABCB1* (PGP) |
| ***ABCC1* (MRP1)** | ***ABCC1* (MRP1)** | *ABCC1* (MRP1) |
| *ABCC2* (MRP2) | *ABCC2* (MRP2) | *ABCC2* (MRP2) |
| ***ABCG2* (BCRP)** | ***ABCG2* (BCRP)** | *ABCG2* (BCRP) |
| *ABCG4* | *ABCG4* | *ABCG4* |
| *SLC2A1* (GLUT1) | *SLC2A1* (GLUT1) | *SLC2A1 (*GLUT1) |
| *SLC22A3* (OCT3) | *SLC22A3* (OCT3) | *SLC22A3* (OCT3) |
| *SLC22A8* (OAT8) | *SLC22A8* (OAT8) | *SLC22A8* (OAT8) |
| *SLC7A5* (LAT1) | *SLC7A5* (LAT1) | *SLC7A5* (LAT1) |
| ***SLCO1A2* (OATP12)** | ***SLCO1A2* (OATP12)** | *SLCO1A2* (OATP12) |
| ***LRP1*** | ***LRP1*** | *LRP1* |

**Table S4:** Explanation: Transporters that are upregulated are shown in = bold/ red, transporters that are downregulated are shown as bold/blue and unchanged ones are in normal text.

**Table S5: Summary of the effects of FUS treatment on gene expression of BBB transporters in Ctrl, *PSEN1*^COR^ and *PSEN1*^AD^ -iBECs.**

|  |  |  |  |  |  |  |
| --- | --- | --- | --- | --- | --- | --- |
| **UT vs FUS^only^ Imm.** | | |  | **UT vs FUS^+MB^ Imm.** | | |
| **CTRL** | ***PSEN1^COR^*** | ***PSEN1*^AD^** |  | **CTRL** | ***PSEN1^COR^*** | ***PSEN1*^AD^** |
| *ABCA1* | *ABCA1* | *ABCA1* |  | *ABCA1* | *ABCA1* | *ABCA1* |
| *ABCB1*  (PGP) | *ABCB1*  (PGP) | ***ABCB1***  **(PGP)** |  | ***ABCB1* (PGP)** | *ABCB1* (PGP) | ***ABCB1 (PGP)*** |
| *ABCC1* (MRP1) | ***ABCC1* (MRP1)** | ***ABCC1* (MRP1)** |  | *ABCC1* (MRP1) | *ABCC1* (MRP1) | ***ABCC1* (MRP1)** |
| *ABCC2* (MRP2) | *ABCC2*  (MRP2) | *ABCC2*  (MRP2) |  | *ABCC2* (MRP2) | *ABCC2*  (MRP2) | *ABCC2*  (MRP2) |
| *ABCG2*  (BCRP) | *ABCG2*  (BCRP) | *ABCG2*  (BCRP) |  | *ABCG2*  (BCRP) | *ABCG2*  (BCRP) | *ABCG2*  (BCRP) |
| ***ABCG4*** | *ABCG4* | *ABCG4* |  | *ABCG4* | *ABCG4* | *ABCG4* |
| *SLC2A1*  (GLUT1) | *SLC2A1*  (GLUT1) | *SLC2A1*  (GLUT1) |  | *SLC2A1* (GLUT1) | *SLC2A1* (GLUT1) | *SLC2A1* (GLUT1) |
| *SLC22A3* (OCT3) | *SLC22A3* (OCT3) | *SLC22A3* (OCT3) |  | *SLC22A3* (OCT3) | *SLC22A3* (OCT3) | *SLC22A3* (OCT3) |
| *SLC22A8* (OAT8) | *SLC22A8* (OAT8) | *SLC22A8* (OAT8) |  | *SLC22A8* (OAT8) | ***SLC22A8* (OAT8)** | *SLC22A8* (OAT8) |
| *SLC7A5* (LAT1) | *SLC7A5* (LAT1) | *SLC7A5* (LAT1) |  | *SLC7A5* (LAT1) | *SLC7A5* (LAT1) | *SLC7A5* (LAT1) |
| *SLCO1A2* (OATP12) | *SLCO1A2* (OATP12) | *SLCO1A2* (OATP12) |  | *SLCO1A2* (OATP12) | *SLCO1A2* (OATP12) | *SLCO1A2* (OATP12) |
| *LRP1* | ***LRP1*** | ***LRP1*** |  | *LRP1* | ***LRP1*** | ***LRP1*** |
|  |  |  |  |  |  |  |
| **UT vs FUS^only^ 24 h** | | |  | **UT vs FUS^+MB^ 24 h** | | |
| **CTRL** | ***PSEN1^COR^*** | ***PSEN1*^AD^** |  | **CTRL** | ***PSEN1^COR^*** | ***PSEN1*^AD^** |
| *ABCA1* | *ABCA1* | *ABCA1* |  | *ABCA1* | *ABCA1* | *ABCA1* |
| *ABCB1*  (PGP) | *ABCB1*  (PGP) | *ABCB1*  (PGP) |  | *ABCB1* (PGP) | *ABCB1* (PGP) | ***ABCB1 (PGP)*** |
| *ABCC1* (MRP1) | *ABCC1*  (MRP1) | *ABCC1*  (MRP1) |  | *ABCC1* (MRP1) | *ABCC1* (MRP1) | *ABCC1* (MRP1) |
| *ABCC2* (MRP2) | *ABCC2*  (MRP2) | *ABCC2*  (MRP2) |  | ***ABCC2* (MRP2)** | *ABCC2*  (MRP2) | *ABCC2*  (MRP2) |
| *ABCG2*  (BCRP) | *ABCG2*  (BCRP) | *ABCG2*  (BCRP) |  | *ABCG2*  (BCRP) | *ABCG2*  (BCRP) | *ABCG2*  (BCRP) |
| *ABCG4* | *ABCG4* | *ABCG4* |  | *ABCG4* | *ABCG4* | *ABCG4* |
| *SLC2A1*  (GLUT1) | *SLC2A1*  (GLUT1) | *SLC2A1*  (GLUT1) |  | *SLC2A1* (GLUT1) | *SLC2A1* (GLUT1) | *SLC2A1* (GLUT1) |
| *SLC22A3* (OCT3) | *SLC22A3* (OCT3) | *SLC22A3* (OCT3) |  | *SLC22A3* (OCT3) | *SLC22A3* (OCT3) | *SLC22A3* (OCT3) |
| *SLC22A8* (OAT8) | *SLC22A8* (OAT8) | *SLC22A8* (OAT8) |  | *SLC22A8* (OAT8) | *SLC22A8* (OAT8) | *SLC22A8* (OAT8) |
| *SLC7A5* (LAT1) | *SLC7A5* (LAT1) | *SLC7A5* (LAT1) |  | *SLC7A5* (LAT1) | *SLC7A5* (LAT1) | *SLC7A5* (LAT1) |
| *SLCO1A2* (OATP12) | *SLCO1A2* (OATP12) | *SLCO1A2* (OATP12) |  | ***SLCO1A2* (OATP12)** | ***SLCO1A2* (OATP12)** | *SLCO1A2* (OATP12) |
| *LRP1* | ***LRP1*** | ***LRP1*** |  | ***LRP1*** | ***LRP1*** | ***LRP1*** |

**Table S5**: Explanation: Transporters that are upregulated are shown in = bold/red, transporters that are downregulated are shown as bold/blue and unchanged ones are in normal text.
